# Paired box 8 facilitates the c-MYC related cell cycle progress in *TP53-*mutation uterine corpus endometrial carcinoma through interaction with DDX5

**DOI:** 10.1038/s41420-022-01072-8

**Published:** 2022-06-07

**Authors:** Ping Qiu, Youkun Jie, Cheng Ma, Huifeng Chen, Yunna Qin, Kaijia Tu, Liqun Wang, Ziyu Zhang

**Affiliations:** 1 Department of Pathology, Jiangxi Maternal & Child Health Hospital, 330006 Nanchang, Jiangxi P. R. China; 2 Department of Reproductive Health, Jiangxi Maternal & Child Health Hospital, 330006 Nanchang, Jiangxi P. R. China; 3 Department of Oncology, Jiangxi Maternal & Child Health Hospital, 330006 Nanchang, Jiangxi P. R. China

**Keywords:** Oncogenesis, Cancer

## Abstract

As a molecular marker of the female reproductive system, Paired Box 8 is widely used in pathological diagnosis of gynecological tumors, but it is not clear whether its expression level is related to the development of uterine corpus endometrial carcinoma and molecular subtype classifications. Here, we show that PAX8 is up-regulated in *TP53* mutation category of UCEC, which is result from the low methylation level of PAX8 in UCEC. We have identified that genes connected to ribosome, lysosome, ribosome biogenesis and cell cycle as PAX8 targets and demonstrate that modulation of the PAX8-DDX5 interaction influences c-MYC related cell cycle and cell growth. Our work defines DDX5 as a critical PAX8 co-factor, places the PAX8-DDX5 interaction in biological context, and highlights PAX8 as a key point for development of novel anti-MYC therapies in *TP53*-mutation UCEC.

## Introduction

Between 1990 and 2019, the global incidence of uterine corpus endometrial cancer (UCEC) increased significantly at a rate of 0.69% per year, accompanied by an increase in mortality [1]. Approximately 80% of patients with early UCEC have a good prognosis, with a 5-year overall survival rate of 95%, compared with 17% for patients with distant disease [2]. Therefore, patients with late and recurrent UCEC are a major treatment challenge. A major diagnostic challenge is to determine which patients with early UCEC are at low and high risk of disease. In the past, Bockman clinical classification and histological classification were used to classify endometrial cancer, but these rough classification methods cannot completely distinguish low-risk and high-risk groups. In 2013, The Cancer Genome Atlas (TCGA) classified UCEC into four categories: POLE ultramutated, microsatellite instability hypermutated (MSI), copy-number low (CNL), and copy-number high (CNH). Among them, CNH (mainly TP53 mutation) patients have the worst prognosis [3]. Therefore, for this type of patients, clinical management need to be taken more aggressively.

Paired box (PAX) family member genes play an important role in embryonic development and tumor neoplasia, and were first discovered in Drosophila [4]. PAX8 is a member of the PAX transcription factor gene family, which is widely expressed in the female reproductive system, so it is often used as a biomarker of the female reproductive system [5]. However, studies have found that PAX8 is abnormally expressed in several kinds of cancers such as thyroid cancer [6], kidney cancer [7], gastric cancer [8] etc. Therefore, PAX8 is considered to be an oncogene, which has the ability to regulate cell proliferation, cell growth, apoptosis and cell migration [9]. In addition, PAX8^+^ expression is associated with poor disease outcome in UCEC [10], but the molecular pathogenesis of PAX8 in UCEC is rarely explored, and its potential role in UCEC is mysterious. In this study, we found that PAX8 can interact with DDX5 protein to regulate downstream gene sets including ribosome, lysosome, ribosome biogenesis, and cell cycle. This may be directly related to the regulation of c-MYC transcriptional activity by PAX8-DDX5. Most importantly, PAX8 has a high expression and low methylation level in the TP53 mutant group UCEC. Through the gain of function experiment, we found that DDX5 and c-MYC can reverse the cell cycle arrest and cell growth slowdown caused by the weakening of PAX8. Based on the above data, it can give us a deeper understanding of the significance of PAX8 in the molecular classification of UCEC and highlights PAX8 as a key point for development of novel anti-c-MYC therapies in TP53-mutation UCEC.

## Results

### PAX8 is highly expressed in uterine corpus endometrial carcinoma

**Fig. 1:**
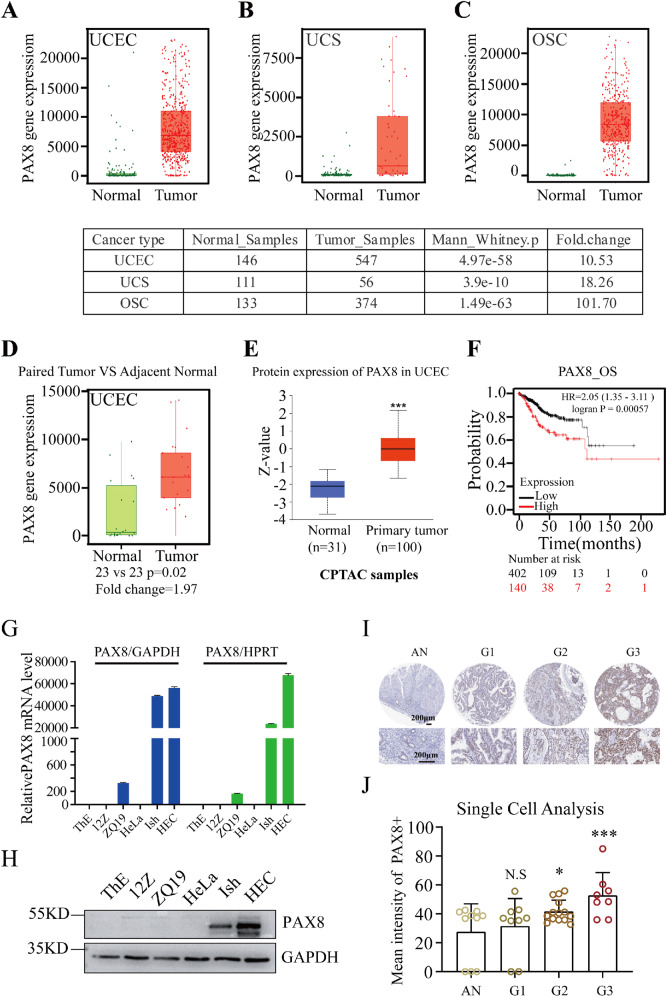
PAX8 overexpression is associated with poor prognosis in UCEC. **A**–**C** PAX8 mRNA levels in normal and cancer tissues of Uterine Corpus Endometrial Carcinoma (UCEC), Uterine Carcinosarcoma (UCS) and Ovarian serous cystadenocarcinoma (OSC) from TNMPLOT online database. **D** The PAX8 gene expression levels between adjacent normal tissue and paired UCEC tissue (TNMPLOT online database). **E** The protein level of PAX8 between normal tissues (*n* = 33) and primary tumor tissues (*n* = 100) from UALCAN online database. Z-values represent standard deviations from the median across samples for the given cancer type. Log2 Spectral count ratio values from CPTAC were first normalized within each sample profile, then normalized across samples. **F** Kaplan–Meier plotter was used to analysis the effect of PAX8 expression on overall survival in patients with endometrial cancer. HR = 2.05 (1.35–3.11), logran *p* = 0.00057. **G**, **H** qRT-PCR and Western blot were used to verify PAX8 mRNA and protein level in cells: ThE, 12Z, ZQ19, HeLa, Ishikawa, HEC-1B respectively. **I** Immunohistochemistry was used to detect the expression of PAX8 in endometrial carcinoma. **J** The relationship between the expression of PAX8 and pathological grade of endometrial carcinoma was analyzed by HistoQuest single-cell analysis (Tissue Gnostics GmbH, Vienna, Austria). *p < 0.05, ***p < 0.001.

We examined PAX8 expression in 22 different types of cancer using TNMPLOT online software (https://tnmplot.com/analysis/) [11] and found relatively high PAX8 mRNA levels in uterine corpus endometrial carcinoma (UECE), uterine carcinosarcoma (UCS) and ovarian serous cystadenocarcinoma (OSC) (Fig. 1A–C and Supplementary Fig. 1). We compared the PAX8 mRNA level between adjacent normal tissues and paired tumors from 23 UCEC patients (Fig. 1D). The PAX8 protein level was also evaluated in Clinical Proteomic Tumor Analysis Consortium (CPTAC) Confirmatory/Discovery dataset [12] by using UALCAN online software [13], PAX8 protein level was elevated in UCEC compared with normal tissue (Fig. 1E). Overall survival was lower in the PAX8_high group than in the PAX8_low group in Kaplan–Meier plotter (Fig. 1F) [14]. To certify these above results from databases, we examined the mRNA and protein levels of PAX8 in various types of cells, including ThESCs (a benign endometrial stromal cell line), 12Z (a benign endometrial epithelial cell line), ZQ19 (a benign primary endometrial stromal cells), HeLa (cervical adenocarcinoma cell line) and two UCEC cell lines (Ishikawa and HEC-1B). Of the examined cell lines, two UCEC cell lines harboring significantly higher mRNA and protein levels of PAX8 than that in other cell lines (Fig. 1G, H). To evaluate PAX8 expression in UCEC tissues, immunohistochemistry (IHC) using an anti-PAX8 antibody was performed in 11 human adjacent normal tissues and 32 UCEC tissues, which were categorized according to their histological grade. Increased PAX8 protein levels were associated with an increasing histological grade (Fig. 1I, J). These data indicates that PAX8 is overexpressed in UCEC, of which is resulted from its high transcriptional level.

### The high methylation level of PAX8 in UCEC is associated with TP53 mutation status

**Fig. 2:**
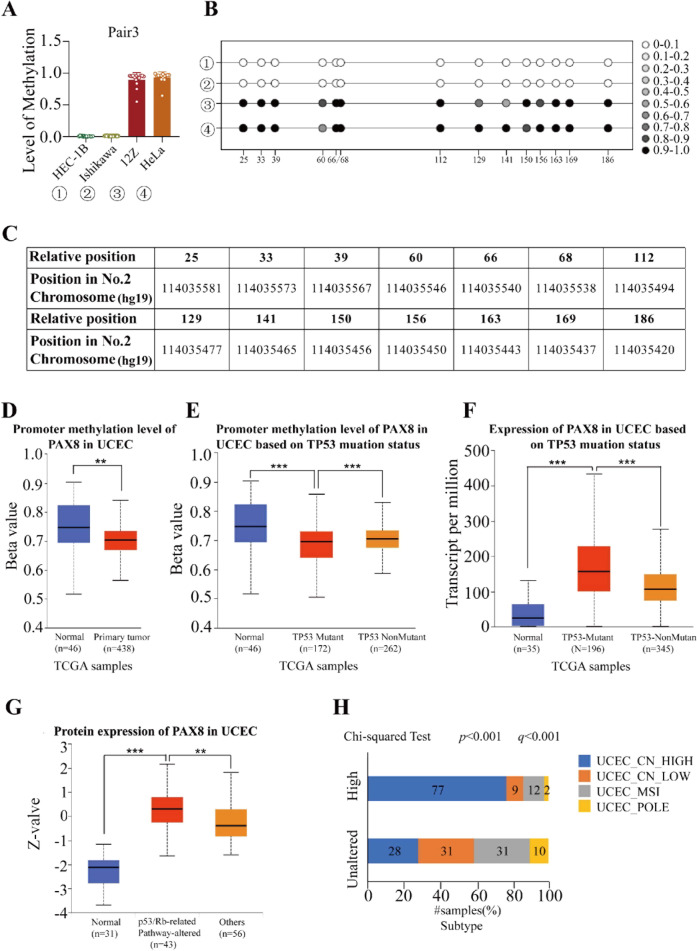
Relationship between methylation level of PAX8 and TP53 mutation (CNH) in molecular subtyping of UCEC. **A** The methylation levels of PAX8 (pair3) in HEC-1B, Ishikawa, 12Z, and HeLa cells were detected by bisulfite conversion sequencing. **B** Schematic diagram of methylation information of all sites in pair3. **C** Statistical table of methylation information for all sites in pair3. **D** Promoter methylation level of PAX8 in UCEC was analyzed by TCGA samples. **E** Promoter methylation level of PAX8 in UCEC based on TP53 muation status was analyzed by TCGA samples. The Beta value indicates level of DNA methylation ranging from 0 (unmethylated) to 1 (fully methylated). **F** Expression of PAX8 in UCEC based on TP53 muation status was analyzed by TCGA samples. **G** Protein level of PAX8 in UCEC based on p53-related pathway. **H** The proportion of PAX8 copy amplification/mRNA upregulation (Altered group) in four molecular subtypes of endometrial cancer. *p < 0.01, **p < 0.005, ***p < 0.001.

DNA methylation can cause gene silencing, so we consider whether the overexpression of PAX8 in endometrial carcinoma may be related to this epigenetic change. For this reason, we first predicted that the PAX8 promoter region has five CpG islands, and then used the bisulfite methylation sequencing (BSP) method to detect the PAX8 promoter methylation level in the four groups of cell lines. It was found that the level of methylation in two endometrial cancer cell lines was significantly lower than that of 12Z (a benign endometrial glandular epithelial cell line) and HeLa (Fig. 2A–C, Supplementary Fig. 2). We found that the methylation level of the PAX8 promoter region in endometrial cancer tissues was also significantly lower than that in the normal control group in the TCGA database (Fig. 2D). Moreover, the promoter methylation level of PAX8 in the endometrial cancer tissues with TP53 mutation was also lower than that in the TP53 non-mutated group (Fig. 2E), and the expression pattern of PAX8 was just the opposite (Fig. 2F). In the tissues with altered p53/Rb pathway, the protein level of PAX8 was significantly higher than that of the normal and other groups (Fig. 2G). It was also found that the proportion of copy-number high (CNH) in samples with high expression of PAX8 was significantly higher than that in the PAX8 unaltered group (Fig. 2H). The above results indicate that the high expression of PAX8 in UCEC is related to the status of PAX8 methylation level, which is associated with TP53 mutation. In addition, we also performed next-generation sequencing (NGS) on the TP53 gene in Ishikawa and HEC-1B cells, respectively, and found that their TP53 genes were exon7c.743 G > A (p.R248Q) and exon7c.736 A > G (p. M246V), and both of them were likely oncogenic [15, 16].

### Knockdown PAX8 influences expression of genes connected to ribosome, lysosome, ribosome biogenesis and cell cycle

**Fig. 3:**
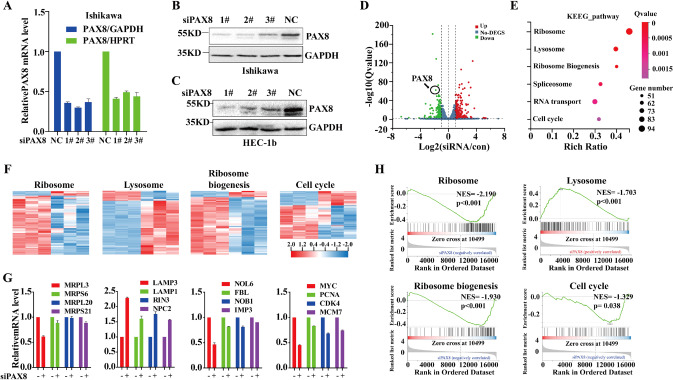
siPAX8 influences the expression of genes involved in ribosome biogenesis and cell cycle pathways. **A** Ishikawa cell was transfected with PAX8 siRNA for 24 h, RNA isolated, and RNA-Seq performed. The expression level of PAX8 mRNA after knockdown PAX8 was detected by qPCR. **B**, **C** The expression level of PAX8 protein was detected by Western blotting, after knockdown PAX8 in Ishikawa and HEC-b cells. **D** Volcano plots of differentially expression genes (red means up-regulated genes, green means down-regulated genes). **E** Categories from the top six families in KEEG pathways analysis of significant gene expression changes under siPAX8. **F** Differentially expressed genes clustering diagram. The columns represent different samples (three groups on the left are the siNC, and the three groups on the right are siPAX8), the rows represent different genes, and are clustered by lg (TPM + 1) value, red indicates high expression genes, and blue indicates low expression genes. **G** qPCR was used to verify ribosome, ribosome synthesis, lysosome, and cell cycle-related gene mRNA expression level in Ishikawa cells after knockdown PAX8. **H** Representative enrichment plots of the ribosome, ribosome synthesis, lysosome, and cell cycle-related gene set which positively or negatively correlate with siPAX8.

Next, in order to further study how PAX8 affects UCEC, we used siRNA to successfully knock down PAX8 in Ishikawa cells and HEC-1B cells (Fig. 3A–C, Supplementary Fig. 3), we used RNA-sequencing (RNA-Seq) to monitor the transcriptomic changes associated with PAX8 siRNA-1# in UCEC cell line Ishikawa. As a result, it was found that a total of 4376 genes were changed, 2245 genes were up-regulated, and 2131 genes were down-regulated (Fig. 3D). KEGG pathway enrichment analysis revealed that transcripts decreased by introduction of siPAX8 are strongly linked to ribosome, ribosome biogenesis, and cell cycle, while those that are induced have links to lysosome (Fig. 3E, F). We also used qPCR to verify some of the different expression genes in the above pathways, and found that the expression of MRPL3 in the ribosome, NOL6 in the ribosome biogenesis pathway and the c-MYC gene in the cell cycle pathway were down-regulated to about 0.5 times after PAX8 knockdown (Fig. 3G). On the contrary, the LAMP3, LAMP1, RIN3, and NPC2 in lysosome could be up-regulated by more than 1.5 times after PAX8 knockdown (Fig. 3G). We found that ribosome, ribosome biogenesis, and cell cycle-related gene sets were less enriched in the PAX8 knockdown group than in the control group, while the lysosomal-related genes were the opposite (Fig. 3H). The changes in the downstream pathway gene set caused by PAX8 knockdown partially overlap with c-MYC induced gene set [17]. These results suggest that PAX8 promotes the development of endometrial cancer by affecting c-MYC.

### C-MYC gene activation is responsible for PAX8 overexpression in UCEC

**Fig. 4:**
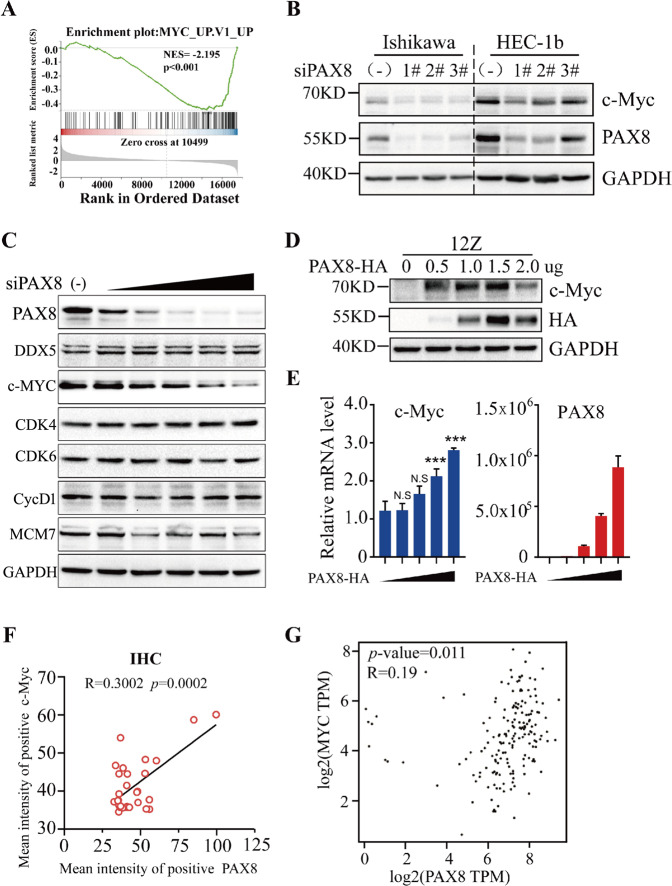
C-MYC gene activation is responsible for PAX8 overexpression in UCEC. **A** Representative enrichment plots of the c-MYC-related gene set which negatively correlate with siPAX8 in Ishikawa cell. **B** PAX8 expression was knocked down by siRNA transfection, cells were collected 48 h after transfection, the expression level of PAX8 and c-MYC proteins in Ishikawa and HEC-1B cells were detected by Western blotting. **C** After gradient knockdown of PAX8 in Ishikawa cells, the expression level of PAX8 and cell cycle-related proteins were detected by Western blotting. **D**, **E** PAX8 was overexpressed by transfected HA-PAX8 plasmid (0ug, 0.5ug, 1ug, 1.5ug, 2ug) in 12Z cells, after 48 h transfection, total RNA and total protein were extracted from cells for qPCR and Western blot analysis, respectively. **F** Linear regression and correlation of PAX8 versus c-MYC protein level. R value is Pearson’s correlation coefficient. **G** The mRNA expression level of c-MYC was positively correlated with PAX8 in GEPIA. N.S. not significantly different among the groups by Student’s t-test. *p < 0.01, **p < 0.005, ***p < 0.001.

In order to further verify the relationship between PAX8 and c-MYC, we found in previous RNA-seq that knockdown of PAX8 is negatively correlated with the up-regulated gene set of c-MYC (Fig. 4A). When PAX8 was knocked down using three different small interfering RNAs (siRNAs), the c-MYC protein level was significantly downregulated in two cell lines, indicating PAX8-dependent expression of c-MYC (Fig. 4B). Gene ontology (GO) analysis revealed that transcripts decreased by siPAX8 are strongly linked to G1/S phase transition in of mitotic cell cycle (Supplementary Fig. 4A and 4B). Moreover, we determined the effect of siPAX8 on the key proteins during G1/S phase transition. Knockdown of PAX8 in HEC-1B cells decreased expression of CDK6, cyclin D1, and MCM7 as well as inhibited c-MYC, while CDK4 expression was unaltered (Fig. 4C). Overexpression of PAX8 in 12Z cells exerted the opposite effect, the protein and mRNA level of c-MYC were both elevated by introducing PAX8 (Fig. 4D, E). Pearson’s correlation analyses revealed a significant positive correlation between protein and mRNA levels of PAX8 and c-MYC (Fig. 4F, G).

### PAX8 interacts with DDX5 in the nucleus

**Fig. 5:**
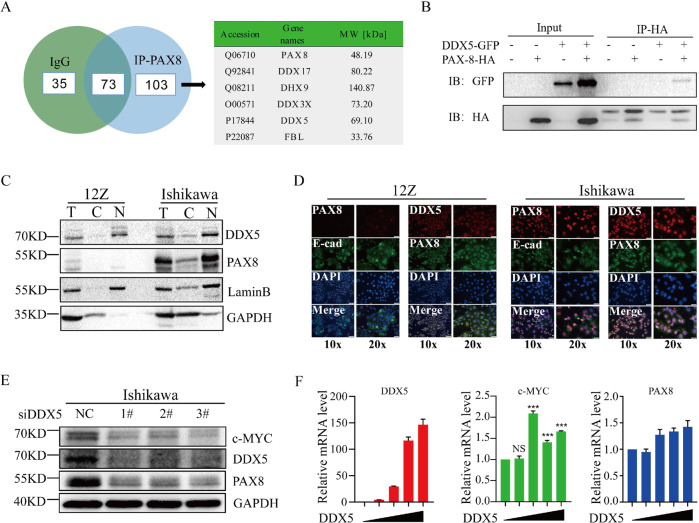
DDX5 is a partner of PAX8. **A** PAX8-interacting proteins in Ishikawa cell were precipitated by using anti-PAX8 antibody, and identified by LC-MS/MS analyses. The proteins identified only in PAX8 precipitates are listed. **B** Co-IP analyses of HA-PAX8 and DDX5-GFP interaction in HEK293 cells. **C** Western analyses of the nuclear and cytoplasmic fractionated samples from 12Z and Ishikawa cells. Lamin B and tubulin were used as the nuclear (N) and cytoplasmic (C) markers, respectively. **D** Immunofluorescence was used to determine the localization of PAX8 and DDX5 in 12Z and Ishikawa cells. DAPI staining was included to visualize the cell nucleus (Blue). Scale bar = 200 μm in 100x and 100μm in 200x vision. **E** PAX8 was knocked down in Ishikawa cells by using siRNA, after 48 h transfection, total protein was extracted from cells for Western blot analysis. **F** Gradient transfection of DDX5 plasmid in 12z cells, 24 h later, RNA was extracted to detect the expression of DDX5, c-MYC, and PAX8, Hprt was used as a housekeeping gene.

Then the molecular mechanism of how PAX8 promotes downstream c-MYC transcription levels remains unclear. Since PAX8 is a transcription factor, its transcriptional activity depends on specific transcriptional co-activators (co-activators). To identify the PAX8-interacting proteins, we immunoprecipitated the HA-tagged PAX8 construct that was overexpressed in HEK293T cell with anti-HA antibody, and found the Co-IP proteins using liquid chromatography-tandem mass spectrometry (LC-MS/MS). After excluding the proteins in the IgG control, we found that DDX5 can interact with PAX8 in the remaining 103 proteins (Fig. 5A). The *DDX5* gene is a member of the DEAD box family that encodes RNA helicases. Due to its role as an adaptor protein, it participates in various cellular processes, including as a transcriptional coactivator. Wu et al proved that DDX5 can bind to the MycG4 structure of the c-MYC promoter region to resolve the MycG4 structure and promote the expression of c-MYC in cancer cells [18]. In addition, the proteins identified in our LC-MS/MS data also include DDX5 binding proteins DDX17, DHX, DDX3X, and FBL (Supplementary Tables 2 and 3), which indicates that PAX8 might be able to form a complex with these proteins through DDX5 (Fig. 5A). In support of this possibility, the interaction between PAX8 and DDX5 was verified by immunoprecipitation and immunoblotting using HEK293T cells co-expressing HA-PAX8 and GFP-DDX5 (Fig. 5B). A biochemical fractionation and immunofluorescent staining experiment showed that both PAX8 and DDX5 were primarily in the nucleus in Ishikawa cell, but not in 12Z cell (Fig. 5C, D).

### C-MYC and DDX5 can rescue the cell growth slowing and cell cycle arrest caused by PAX8 knockdown

**Fig. 6:**
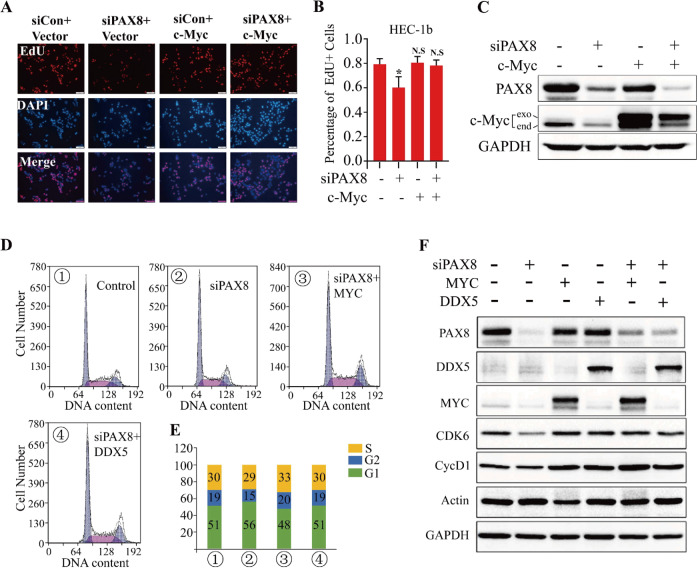
PAX8 promotes UCEC cell growth and cell cycle progress dependent on DDX5 and c-MYC in vitro. **A** EdU incorporation assays of HEC-1b cell transiently transfected with siNC or siPAX8 or c-MYC or siPAX8 + c-MYC. **B** The percentage of EdU positive cells was blindly calculated with counting nine nonoverlaping fields. Values are means ± s.d. **C** HEC-1B cells were transfected with siRNAs or plasmid to knockdown PAX8 or overexpression c-MYC and DDX5, respectively. The proteins were extracted from cells for Western blotting analysis. **D**, **E** HEC-1B cells were transfected with plasmid or siRNA to knockdown PAX8 and overexpression c-MYC and DDX5, after treatment for 48 h. Cells were assayed by flow cytometry. The percentages of the cells in G1, S, and G2 phase were counted and compared. **F** HEC-1b cells were transfected with siPAX8 or siPAX8 + c-MYC or siPAX8 + DDX5. Representative G1/S transition markers were immunoblotted.

Based on the above results, can DDX5 also participate in the regulation of c-MYC expression? Therefore, we first knocked down DDX5 in endometrial cancer cells by siRNA, and found that the protein expressions of both c-MYC and PAX8 were reduced (Fig. 5E). In addition, after overexpressing DDX5 in 12Z cells, it was found that the mRNA level of c-MYC was significantly increased, but the mRNA of PAX8 was not changed much (Fig. 5F). To further explore the function of DDX5 and PAX8 on endometrial cancer cells, knockdown of PAX8 expression decreased the proliferation of both HEC-1B and Ishikawa cells relative to that of control cells, and reconstitution of c-MYC restored the proliferation of these cells (Fig. 6A–C, Supplementary Fig. 5), which also verifies that c-MYC is a downstream gene of PAX8. The results of cell cycle experiments showed that knocking down PAX8 could increase the G1 phase, while rescue of DDX5 and c-MYC could restore to the control level (Fig. 6D), indicating that PAX8 regulates cell cycle arrest in the G1/S phase and is dependent on DDX5 and c-MYC. Since PAX8 has an effect on G1/S transformation, whether it is involved in the regulation of cell cycle-related proteins, we found that siPAX8 can significantly inhibit the protein levels of CDK6 and CycD1, and rescue c-MYC and DDX5 can restore the expression of these two proteins to the siRNA control group (Fig. 6E).

## Discussion

With the development of molecular classification of endometrial cancer, the precision treatment of endometrial cancer has become more personalized. In particular, patients diagnosed with abnormal TP53 have a poor prognosis, so the subclassification of this group of patients is important. PAX8 has been used for a long time as a marker to distinguish whether it is derived from the female reproductive system, but its role in tumor progression seems to have been neglected. We detected the expression of PAX8 in endometrial cancer from mRNA to protein multidimensional by using the results from related databases, western blot, immunohistochemistry, and qPCR (Fig. 1), and found that compared with the normal control, PAX8 was indeed overexpressed in endometrial cancer and was directly correlated with prognosis (Fig. 1F). Here, we used a benign endometrial glandular epithelial cell line 12Z, in which the expression of PAX8 was much lower than that of cancer cells, and in primary stromal cells ZQ19 from patients with endometriosis, we found that the expression of PAX8 was also higher than that of control stromal cells ThESCs. These results suggest that PAX8 may play an important role not only in the progression of endometrial cancer, but also in the development of endometriosis.

So why is PAX8 highly expressed in endometrial cancer cells? Wu et al. reported that the activation of PAX2 gene in endometrial carcinoma is caused by low-level methylation of its promoter region [19]. In addition, Shi et al. verified that HDAC inhibitors can reduce the expression of PAX8 and related genes through epigenetic changes [20]. Based on this, *Methprimer* software was used to predict six CpG islands in the PAX8 promoter region [21]. Bisulfate-based amplicon sequencing (BSAS) technique revealed that methylation of the PAX8 promoter region in the two endometrial cancer cells was significantly lower than that in the control cells (Fig. 2A–C, Supplementary Fig. 2). HeLa was selected because it was originally a glandular epithelial tumor cell from the female reproductive system, while 12Z cell was selected as a control cell because it was a benign endometrial glandular epithelial cell. According to the above results, the promoter hypomethylation level of PAX8 is highly specific in endometrial cancer, which can be used as a potential screening method for endometrial cancer in the future. Tovy et al. reported that p53 contributes to maintaining DNA methylation homeostasis and tumor suppressor function by balancing the expression of methyltransferase Dnmt3 family and demethylase Tet [22]. Therefore, mutation of TP53 gene is bound to lead to disorder and instability of genome methylation. To this end, we used TCGA database to observe the relationship between PAX8 methylation level and TP53 mutation in real human tissues. We found that the methylation level of PAX8 in endometrial carcinoma tissues was significantly lower than that of normal controls, and the TP53 mutant samples also showed the lowest methylation level and the highest transcription level (Fig. 2D–F), and this kind of tendency was consistent with changes in p53-related pathways (Fig. 2G). Similarly, the proportion of endometrial cancer molecular typing TP53 mutations was significantly increased in samples with high PAX8 expression level (77% vs 28%, Fig. 2H).

As a transcription factor, PAX8 is bound to regulate the transcriptional activity of some downstream genes. We used RNA-Seq technology to explore the changes in the downstream regulatory gene network caused by PAX8 knockdown. Indeed, we found that knocking down PAX8 up-regulated 2245 genes and down-regulated 2131 genes. A large number of cell cycle-related genes were changed (Fig. 3E), and most of the pathway-related genes were down-regulated (Fig. 3F), indicating that siPAX8 obstructed ribosome synthesis and cell cycle progress. Adler et al. used ChIP-seq to analyze the PAX8 enrichment region in ovarian cancer cells and found that it was closely related to cell mitosis and cell cycle [23]. Ruiz-Llorente et al. also used ChIP-seq to detect the enrichment region of PAX8 in thyroid cells and found that PAX8 was enriched in cell cycle, metabolism, and nucleic acid processing [24], suggesting that PAX8 regulation of downstream cell cycle-related genes may be caused by its regulatory motif binding. More importantly, c-MYC, as an oncogene and a key regulator of cell cycle, was significantly down-regulated after PAX8 knockdown (Figs. 3G and 4B), and the expression of cell cycle-related proteins CDK6, CyclD1, and MCM7 also decreased with PAX8 knockdown (Fig. 4C). Immunohistochemistry and mRNA analysis in the database showed a positive correlation (Fig. 4 and 4G). Gradient overexpression of PAX8 caused c-MYC protein and RNA expression to vary by concentration gradient in benign endometrial glandular epithelial 12Z cells (Fig. 4D, E). GSEA data analysis also revealed that c-MYC-related gene expression was correlated with siPAX8 (Fig. 4A).

**Fig. 7:**
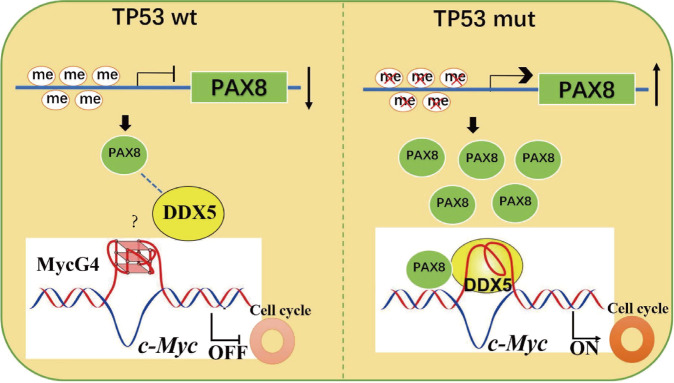
Schematic diagram of the regulation of c-MYC by PAX8. In TP53 wild-type cells, a small amount of PAX8 is insufficient to co-regulate the transcriptional level of c-MYC with DDX5, while in TP53-mutated cells, promoter hypomethylation leads to high expression of PAX8, which in turn cooperates with DDX5, possibly by unraveling the Myc4G structure leading to increased c-MYC expression and promotes cell cycle progression.

Above results demonstrated the involvement of PAX8 in the regulation of C-MYC and its downstream cell cycle pathway. However, is PAX8 directly involved in the expression of c-MYC? To this end, PAX8 antibody was used for ChIP-seq analysis, and it was found that PAX8-enriched DNA fragment not only contained the motif of PAX protein family, but also included several cell cycle-related genes, including Cyclin D, MCM7 and CDK6, however, no enrichment region was found around c-MYC gene (data not shown), which suggests that PAX8 may not be directly involved in the transcriptional regulation of c-MYC, either through indirect enrichment (the enrichment amount is too low to be measured) or through other intermediate factors. The reasons for selecting DDX5 from many binding proteins are as follows: (1) DDX5 can participate in unfolding the G-quadruplexes (G4) structure of c-MYC promoter region, thus promoting its transcriptional activity [18]; (2) DDX5 can form complex with multiple proteins (DDX3, DDX17, DHX9, and FBL) to participate in RNA-independent gene expression regulation [25–27], and these proteins were found in our mass spectrometry data; (3) The interaction between DDX5 and PAX8 in the nucleus was verified by co-IP and immunofluorescence (Fig. 5B–D). More importantly, cell cycle arrest and proliferation retardation caused by PAX8 knockdown could be rescued by DDX5 (Fig. 6). These results indicate that DDX5 is not only downstream of PAX8, but might acts as a transcriptional coactivator of DDX5, indirectly regulating the transcriptional activity of c-MYC (Fig. 7).

## Methods and materials

### Cell lines and culture

Ishikawa and 293 T were maintained in Dulbecco’s Modified Eagle’s medium (BOSTER; China), cell lines HEC-1b and 12Z were grown in MEM medium (Gibco; Thermo Fisher Scientific, USA). All media were contained 10% fetal calf serum and 1% penicillin streptomycin. The cells were maintained in humified incubator-under standard conditions at 37 °C, 5% CO_2_. The confluence of the adherent cells was reached 90–95%, and passaged using 0.25% trypsin-EDTA. Cells within 15–20 passages were used in all experiments.

### RT­qPCR

Total RNA was extracted from cells by using TRIzol reagent (TaKaRa, Dalian, China), then converted to cDNA through the cDNA synthesis kit (TaKaRa, Dalian, China), above experiments were performed according to the manufacturer’s instructions. Reverse transcription was performed using 1 μg cDNA, according to the manufacturer’s protocol. Gene expression was analyzed and quantified by real-time PCR using specific primers. Results were normalized to the expression of GAPDH/HPRT. Data analysis formulas: ΔCt (Control group) = Ct (Objective) − Ct (Internal reference), ΔCt (experimental group) = Ct (Objective) − Ct (Internal reference), ΔΔCt = ΔCt (experimental group)−ΔCt (Control group), ratio (experimental group/control group) = 2 − ΔΔ Ct. The information of primer can be found in Supplementary Table 1.

### Western blot

All experiments total protein was extracted from cells by using RIPA lysis buffer (Beyotime, Shanghai, China). The concentration of total protein from cells was measured by the method of the bicinchoninic acid (BCA) Protein Assay (Beyotime), separated on 10% SDS–polyacrylamide gels. For cells, equal amount of protein was separated on 10% SDS–polyacrylamide gels. The Western blot experiments were carried out according to the routing experimental steps. The protein was transferred to the polyvinylidene membranes (PVDF), then primary antibodies immunoblotted with PVDF, primary antibodies including anti-PAX8 rabbit monoclonal antibody (1:1000, Abcam), anti-PAX8 mouse monoclonal antibody (1:2000, Proteintech), anti-DDX5 rabbit polyclonal antibody (1:200, Proteintech), anti-c-MYC mouse polyclonal antibody(1:2000, Proteintech), anti-Lamin B1 rabbit polyclonal antibody (1:2000, Proteintech), anti-HA tag rabbit polyclonal antibody (1:2000, Proteintech), anti-GFP tag rabbit polyclonal antibody (1:2000, Proteintech), anti-Cyclin D1 mouse monoclonal antibody (1:10000, Proteintech), anti-CDK6 mouse monoclonal antibody (1:10000, Proteintech), anti-CDK4 rabbit polyclonal antibody (1:1000, Proteintech), anti-MCM7 rabbit polyclonal antibody (1:2000, Proteintech), anti-GAPDH mouse polyclonal antibody (1:5000, Santa Cruz Biotechnology), anti-β-actin (ACTB) rabbit polyclonal antibody (1:1000, Boster, Wuhan, China). After washing with PBST, the membranes were incubated with corresponding secondary antibodies (1:3000, Boster). Chemiluminescent reaction (ECL luminescence reagent, Absin Bioscience Inc) was used for detected the bands. GAPDH and ACTB were used as internal reference.

### Small interfering RNA (siRNA) transfection

Ishikawa, HEC-1b, and 12Z cells were transfected with siRNAs by using Lipofectamine^®^ RNAi MAX Reagent (Invitrogen), performed with the manufacturer’s protocol. Negative control siRNA (si-NC), three individual PAX8 siRNAs (si-PAX8 123 1#, 2#, and 3#) were purchased from Invitrogen, and three individual DDX5 siRNAs (si-DDX5123 1#, 2#, and 3#) were purchased from RiboBio (Guang Zhou, China). Briefly, 7.5 μl Lipofectamine^®^ RNAi MAX Reagent was mixed with 25 pmol siRNA duplexes to form complexes at room temperature. At a cell density of 70–80%, cultured cells were transfected with siRNA (si-PAX8, DDX5 or NC). After 48 h transfection, total RNA and total protein were extracted from cells for qRT-PCR and Western blot analysis, respectively.

### Immunofluorescence

Immunofluorescence studies were done according to a standard protocol. Briefly, Ishikawa and 12Z cells were seeded to 24-well plates, reached 75–80% confluency. Cells were washed three times in PBS, medium and non-adherent cells were removed. Adherent cells were fixed in 4% paraformaldehyde for 10 min, then permeabilization with 0.1% Triton X-100 for 10 min. Incubation in 0.5% BSA in PBS at room temperature was used for blocked non-specific sites for 60 min. The primary antibodies used were PAX8 (Abcam, 1:200, SP348), E-cadherin (BD Biosciences, 1:200, 610181), DDX5 (Proteintech, 1:200, 10804-1-AP). Cells were incubated in primary antibodies diluted with blocking buffer overnight at 4 °C. On the second day, cells were washed three times, unbound-antibody were removed, then either Alexa Fluor 488 or 568 conjugated goat anti-rabbit IgG were used for incubated in blocking buffer for 60 min at room temperature protected from light. Cells were incubated for 10 min by using diluted Hoechst. Finally, images were acquired using fluorescent microscope.

### Nuclear and cytoplasmic fraction

To determine the localization of PAX8 and DDX5 in the cytoplasmic nucleus in this study. Ishikawa, HEC-1b, and 12Z were seeded in 6-well plates, reached 90-95% confluency and collected. Nuclear and Cytoplasmic Protein Extraction Kit (Beyotime Institute of Biotechnology, Jiangsu, China) was used to isolate nuclear protein, according to the manufacturer’s instructions. Nuclear and cytoplasmic protein were collected for Western blot analysis.

### Cell proliferation assay

5-ethynyl-2-deoxyuridine (EdU) Labeling/Detection Kit (RiboBio, Guang Zhou, China) was used to assess proliferating cells, according to the manufacturer’s instructions. Ishikawa/HEC-1b cells follow by a density of 1.0 × 10^5^ cells per well were seeded in 24-well plates and, cultured for 16 h. Plasmid DNA or siRNA were transfected cells using appropriate transfection reagents. After 48 h of transfection, the solution of an EdU-labeling kit was diluted in proportion to 1:3000 in cell medium, cells were incubated 18 h at 37 °C under 5% CO_2_. On the second day, cells were treatment with 4% paraformaldehyde and 0.5% Triton X-100, stained with anti-EdU working solution. DAPI was used to label cell nuclei. Images were acquired using a Fluorescence microscope.

### Flow cytometric analysis

For cell cycle analysis, Ishikawa and HEC-1b cells were transfected with plasmid DNA or siRNA treatment for 48 h, collected and stained by using a cell cycle kit (Shanhai BestBio, China). Cells were assayed by flow cytometry. The percentages of the cells in G_0_–G_1_, S, and G_2_–M phase were counted and compared.

### RNA-seq

To explore changes in downstream genes and pathways following knockdown of PAX8 in endometrial cancer cells. RNA-seq was performed at the High throughput laboratory, Huada Medical Laboratory Ltd, Wuhan, China. Cells were resuspended in 1 ml TRIzol (TIANGEN, DP424), and RNA was prepared according to the manufacturer’s instructions. For knockdown PAX8 Ishikawa cells, cells were treated with siRNA for 48 h and harvested. Prepared RNA was submitted to the High throughput laboratory-Huada Medical Laboratory Ltd for DNase treatment, rRNA depletion, library preparation, and 150 bp paired-end sequencing on Illumina HiSeq. Genes were selected and Customized primers based on sequencing results for validation of RNA-seq by reverse transcriptase qPCR (RT-qPCR). Three technical replicates were performed for each sample, and fold changes in gene expression was calculated according to the Relative quantitation method (2 − ΔΔCt) using GAPDH/HPRT as reference gene. Primer sequences used are listed in Supplementary Table 1. Pathway enrichment analysis was performed with the web application Metascape, using a custom analysis including “GO Molecular Function”, “GO Biological Processes”, and “KEGG Pathway” with the default parameters.

### In-gel digestion

To investigate what protein interacts with PAX8, so protein samples were collected for mass spectrometry analysis. For in-gel tryptic digestion, the gel blocks were decolorized in 50% acetonitrile (v/v) with 50 mM NH_4_HCO_3_ until clarified. The gel pieces were dehydrated with 100 μl 100% acetonitrile for 5 min to remove the liquid, rehydrated in 10 mM dithiothreitol, incubated at 56 °C for 60 min. The gel pieces were again dehydrated in 100% acetonitrile to remove the liquid and rehydrated with 55 mm iodoacetamide. Samples were incubated in the dark for 45 min at room temperature. The gels were washed with 50 mM NH_4_HCO_3_ and dehydrated with 100% acetonitrile. The gel pieces were resuspended with 10 ng/μl trypsin in 50 mM NH_4_HCO_3_ for 1 h on ice. Excess liquid was removed, then the gel pieces were digested with trypsin overnight at 37 °C. The peptides were extracted with 50% acetonitrile/5% formic acid, followed by 100% acetonitrile. The peptides were dried to completion, then resuspended in 2% acetonitrile/0.1% formic acid.

### LC-MS/MS analysis

Trypsin peptides were dissolved in 0.1% formic acid (solvent A) and loaded directly onto a home-made reversed-phase analytical column (15-cm length, 75μm i.d.). The gradient was increased from 6% to 23% of solvent B (0.1% of acetonitrile 98%) over 16 min, 23% to 35% in 8 min to climb to 80% in 3 min, then holding 80% of the last 3 min, all at a constant flow rate of 400 NL/min for the EASY-NLC 1000 UPLC system. The peptides were detected by NSI source and coupled with UPLC by Q Exactive^TM^ Plus (Thermo) tandem mass spectrometry (MS/MS). The electrospray voltage applied was 2.0 kV. The m/Z scan range of the full scan was 350–1800, and complete peptides were detected in Orbitrap with a resolution of 70,000. Then the peptides were selected for mass/mass spectrometry analysis using an NCE setting of 28 and detected in Orbitrap at a resolution of 17500. A data-dependent process that alternates between one MS scan and 20 MS/MS scans and includes 15.0 s of dynamic exclusion. Automatic gain control (AGC) is set at 5E4.

### Co-immunoprecipitation

The interaction between PAX8 and DDX5 was verified by transfection of plasmids into cells. 293 T cells were seeded in 6-well plates at a density of 1.0 × 10^5^ cells per well and cultured for 18 h. Upon reaching 70–80% confluence, plasmid PAX8 and DDX5 were transfected to 293 T cells by the GBfectene–Elite method (GB1001-250, Genebank Biosciences Inc), respectively. 24 h after transfection cells were collected for experiment. The antibody was incubated with the beads to capture the immune complex, or deposited on the beads where the antibody binding protein was fixed, and any protein not deposited on the beads were washed away. the antigen was eluted from the support and analyzed by sodium dodecyl sulfate-polyacrylamide gel electrophoresis (SDS-PAGE). Western blot for verified the identity of the antigen.

### Immunochemistry

Immunohistochemistry (IHC) was performed as previously described [28]. The tissue microarray (Cat. HUteA045PG01) of UCEC was purchased from Biobank Center of National Engineering Center for Biochip at Shanghai. The paraffin-embedded tissue microarray was baked in the oven at 65 °C for 12 h. After deparaffinization and blocking, the antigen–antibody reaction was incubated overnight at 4 °C. The primary anti-PAX8 rabbit monoclonal antibody (Cat No: ab227707, Abcam) was used at a dilution of 1:500, anti-c-MYC rabbit polyclonal antibody (Cat No: 10828-1-AP, Proteintech) was used at a dilution of 1:2000. The quantification Images was performed with HistoQuest (Tissue Gnostics GmbH, Vienna, Austria, www.tissuegnostics.com).

### Statistical analysis

Data are expressed as mean ± SD or SEM. Statistical significance was determined using the student’s t-test (two-tailed) or one-way ANOVA. *P* < 0.05 was considered statistically significant. Mann-Whitney U test was used to analyze the expression level of PAX8 in two independent groups. The Kaplan–Meier method test was utilized for progression-free survival (PFS) and overall survival (OS) analysis. Correlation of PAX8 expression with c-MYC expression in UCEC cells was analyzed by Pearson correlation test. Chi-square test was used to analyze the proportion of PAX8 high expression samples in UCEC molecular subtyping. Unless otherwise stated, all experiments were done at least three independent, biological replicates, and statistical tests were carried out in PRISM 9 (GraphPad).

## Supplementary information

Original western blot data

SUPPLEMENTAL MATERIAL

## Data Availability

The datasets generated during the current study are available from the corresponding author on reasonable request.
