# Supplementary material for: Paired box 8 facilitates the c-MYC related cell cycle progress in TP53-mutation uterine corpus endometrial carcinoma through interaction with DDX5
Source: Cell Death Discov. 2022 Jun 7;8:276. doi: 10.1038/s41420-022-01072-8 (PMC9174161; doi:10.1038/s41420-022-01072-8)
Supplement: Supplementary file 1 — Original western blot data [file 41420_2022_1072_MOESM1_ESM.pdf]

Original western blotting data

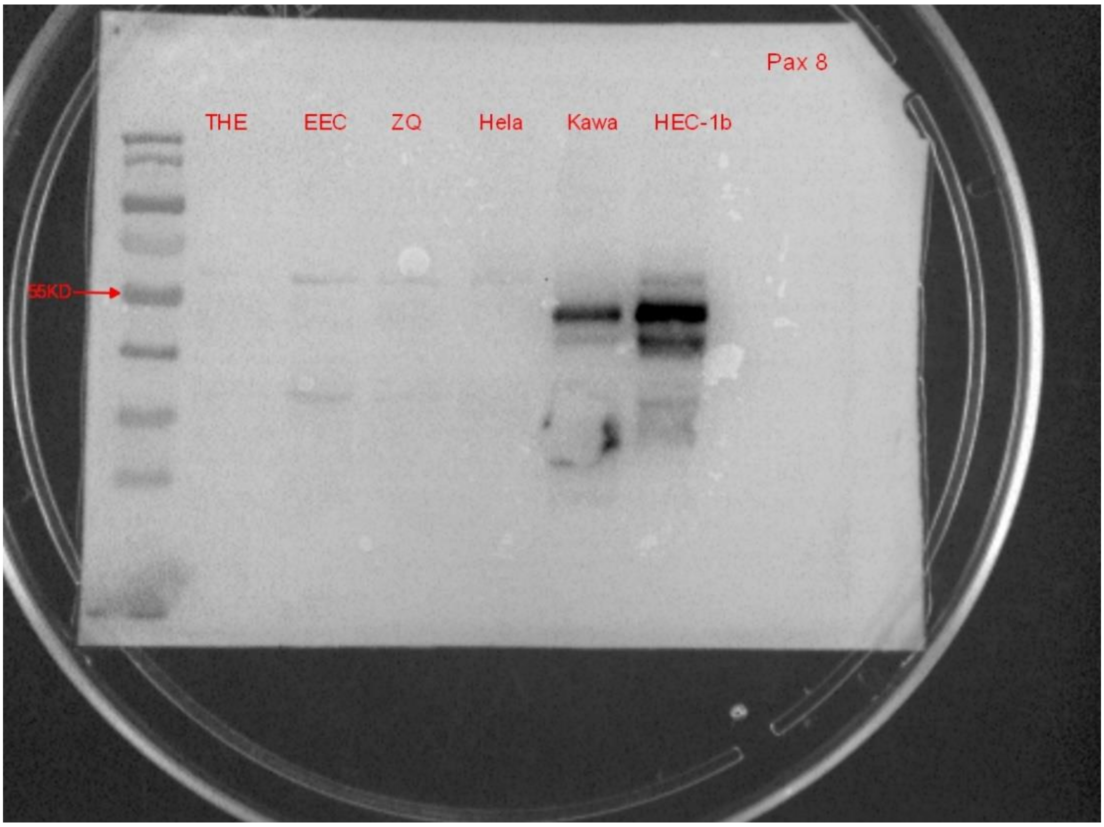

PAX8

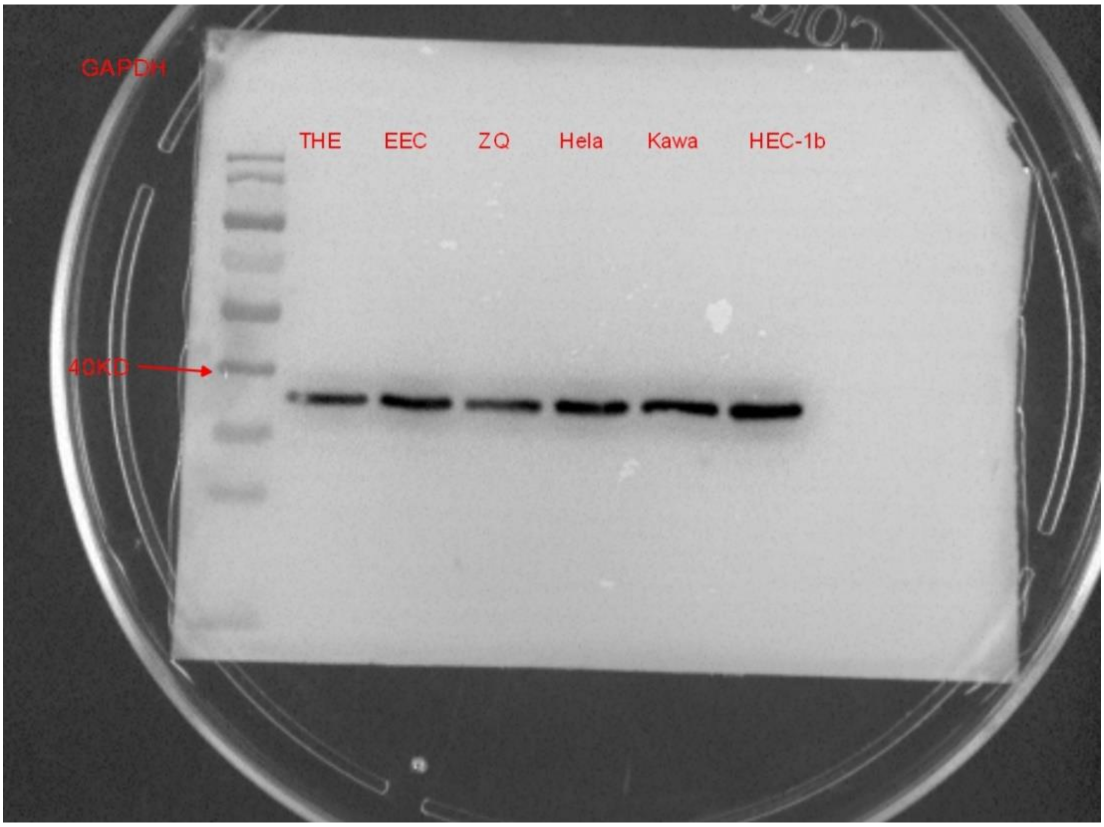

GAPDH

*Original western blotting data(Figure1 H).

Original western blotting data

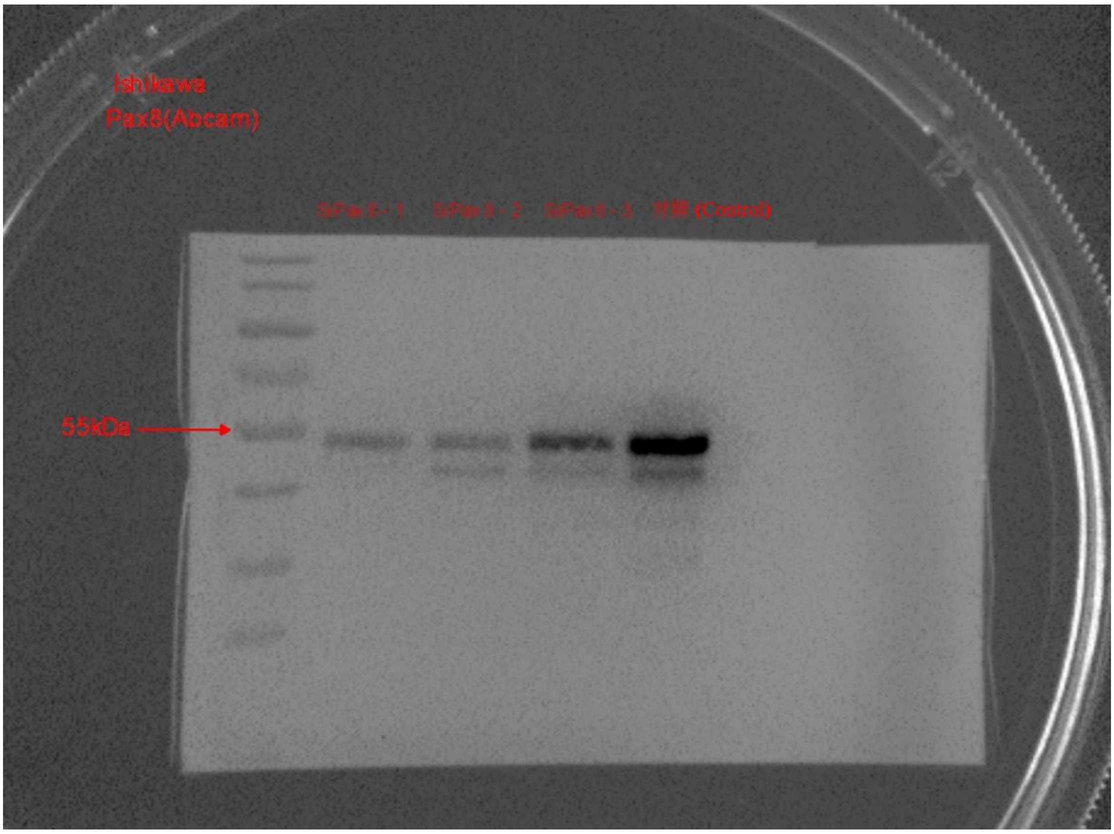

PAX8

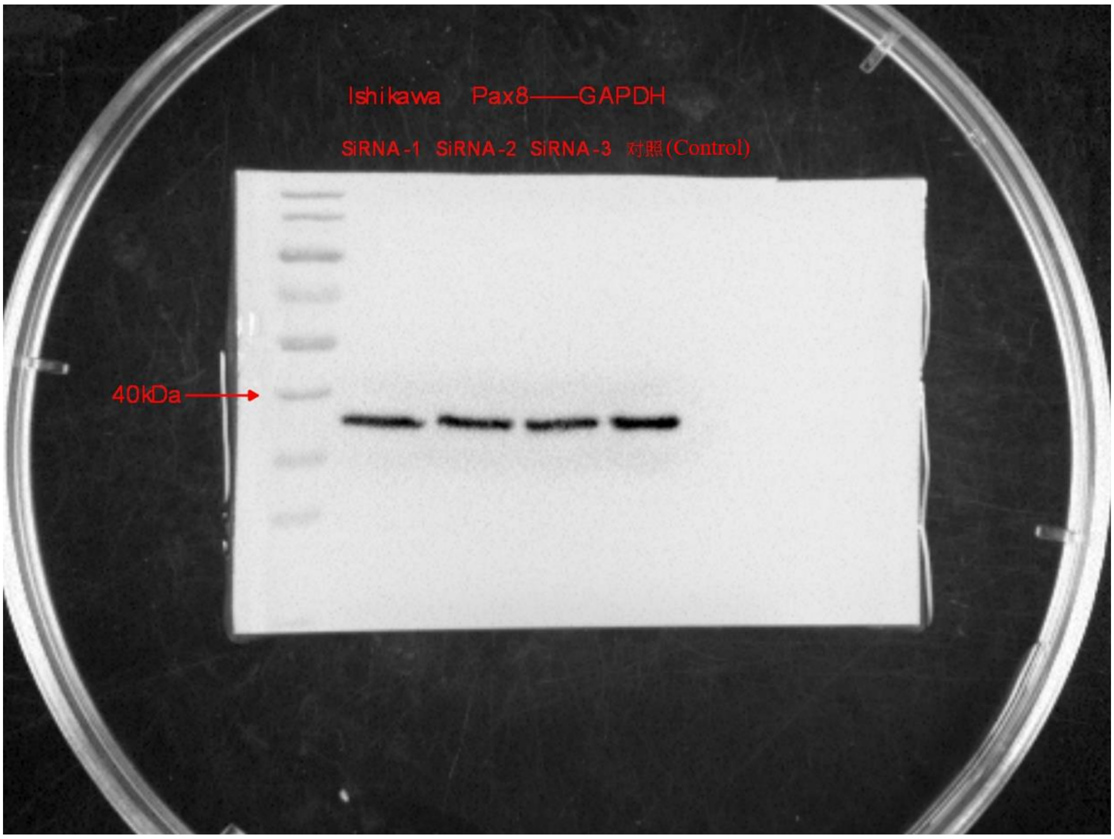

GAPDH

*Original western blotting data(Figure3 B).

Original western blotting data

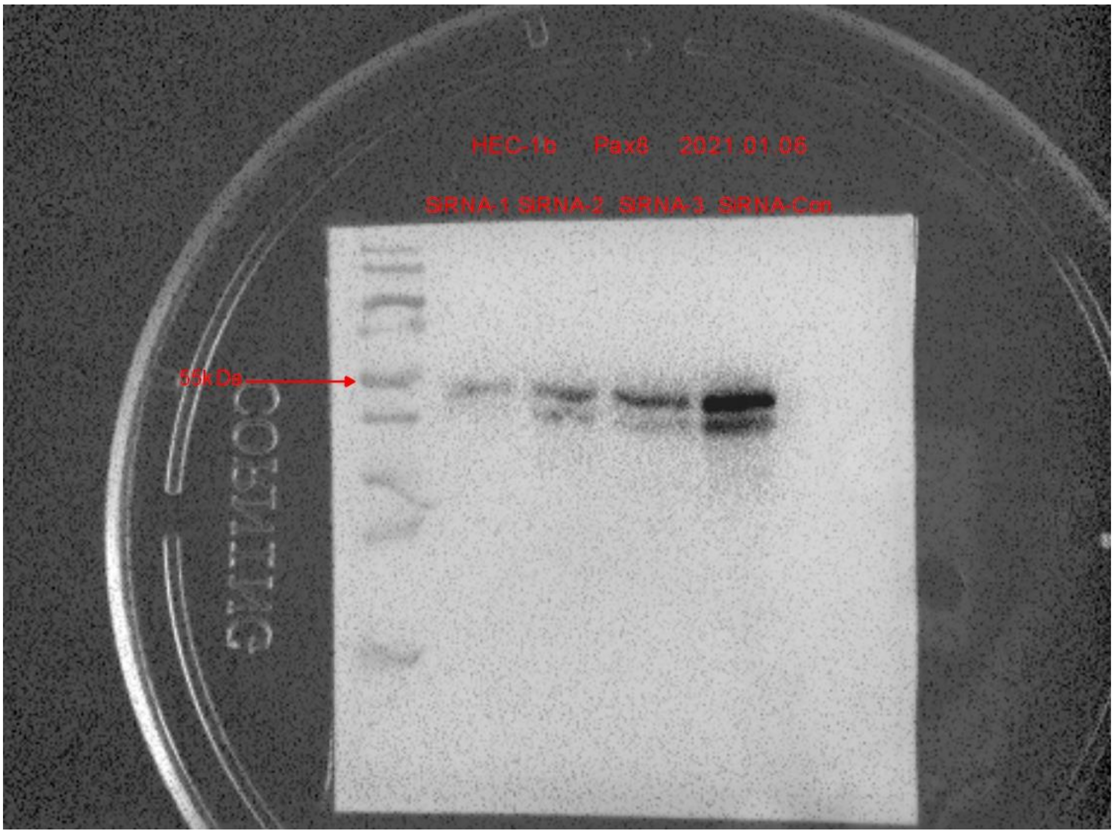

PAX8

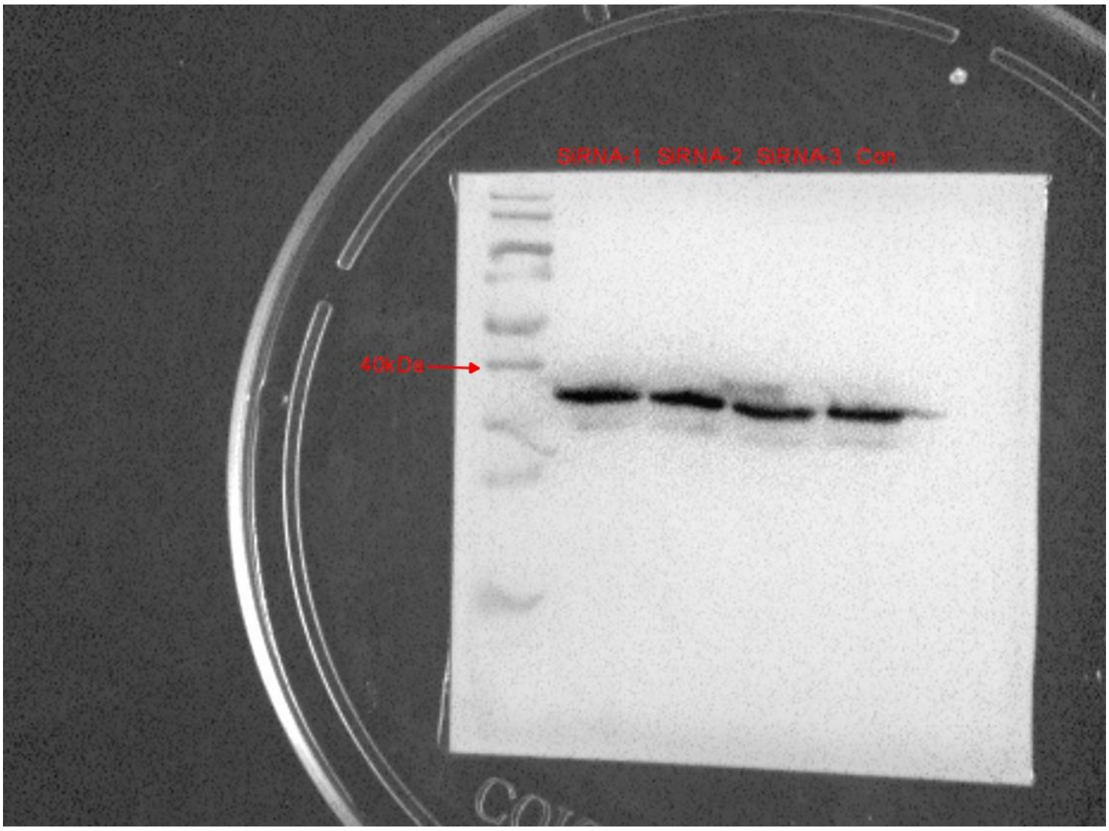

GAPDH

*Original western blotting data(Figure3 C).

Original western blotting data

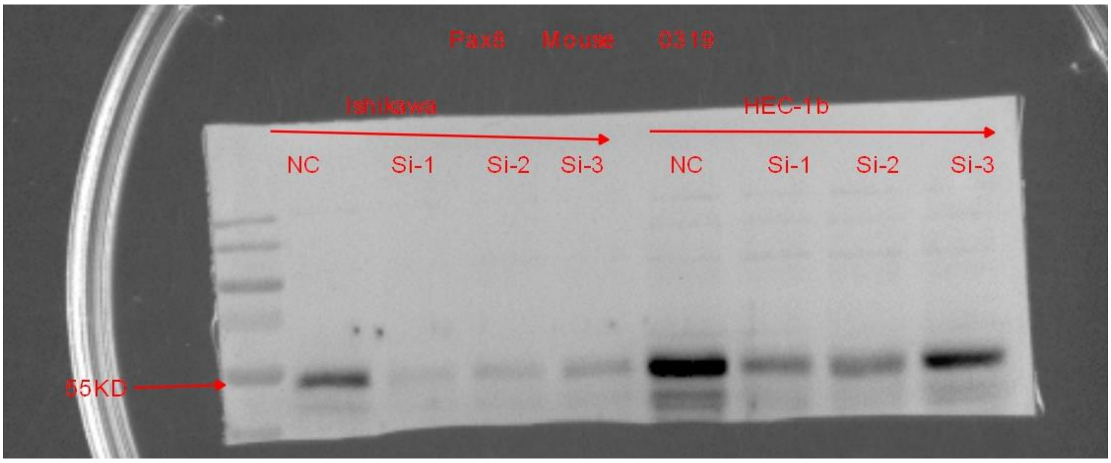

PAX8

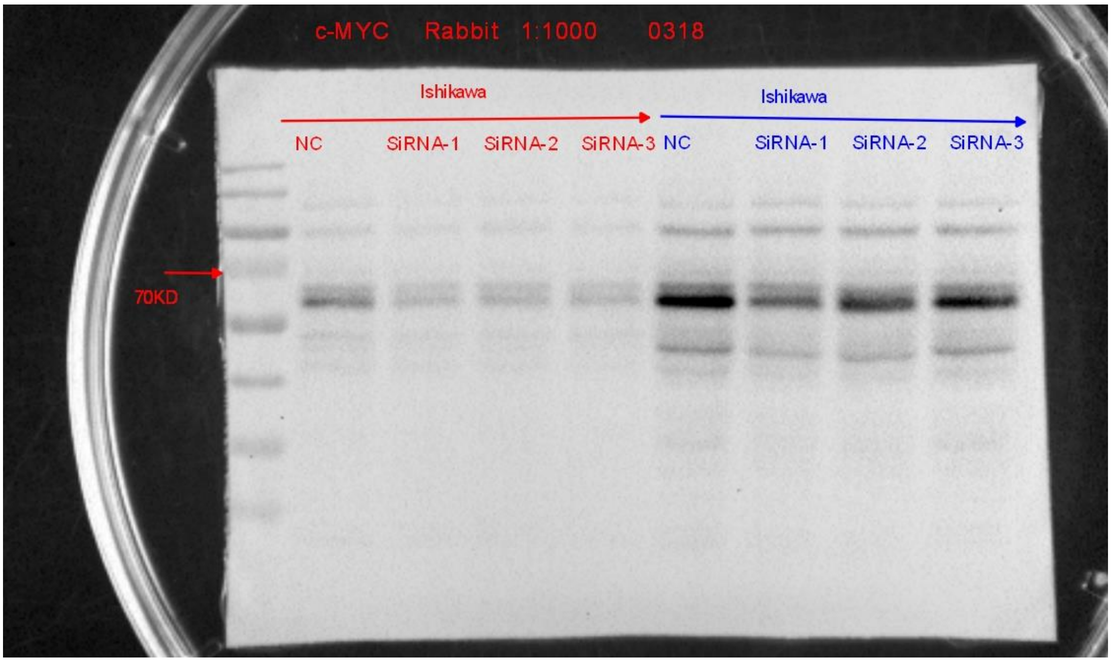

c-MYC

Original western blotting data

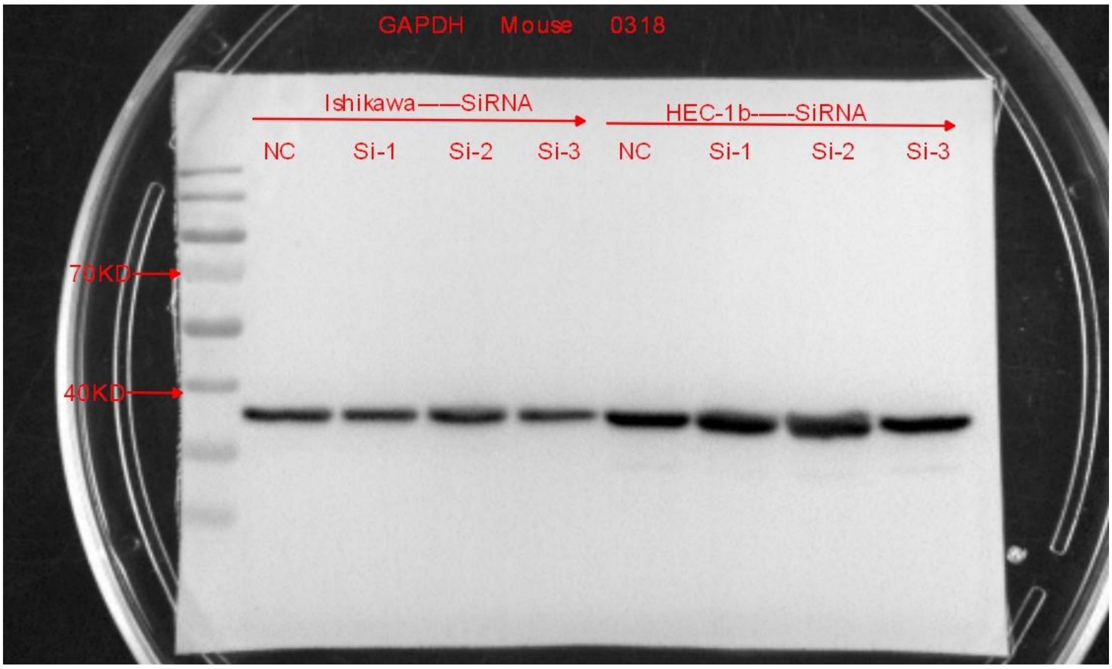

GAPDH

*Original western blotting data(Figure4 B).

Original western blotting data

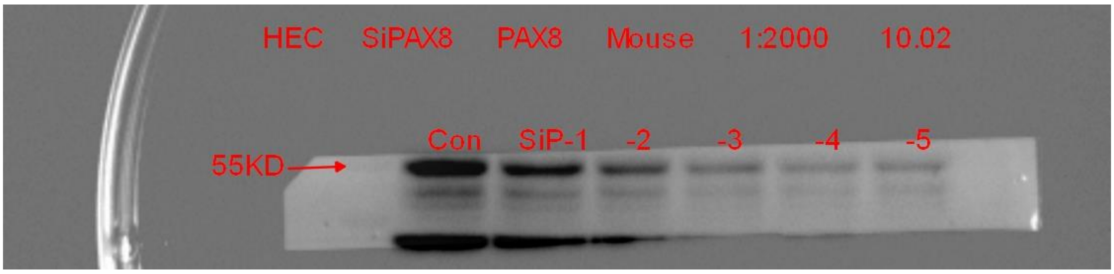

PAX8

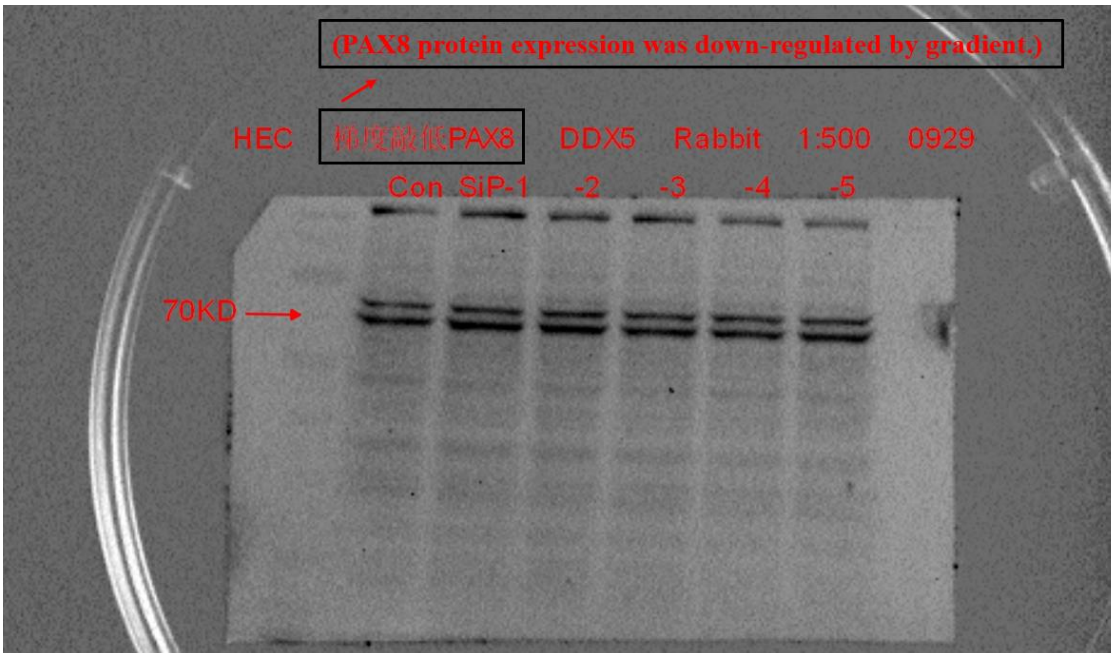

DDX5

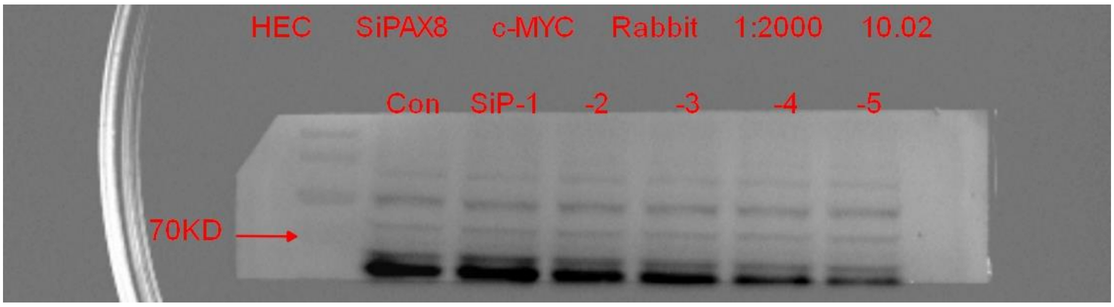

c-MYC

Original western blotting data

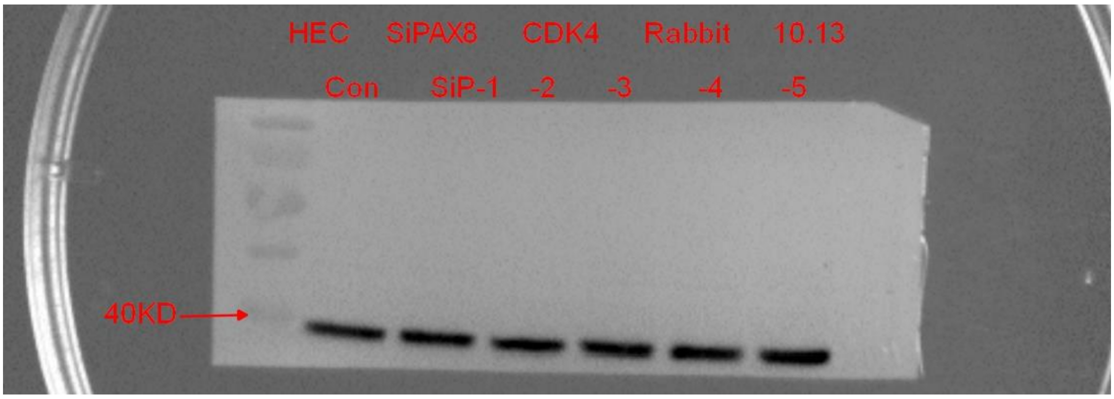

CDK4

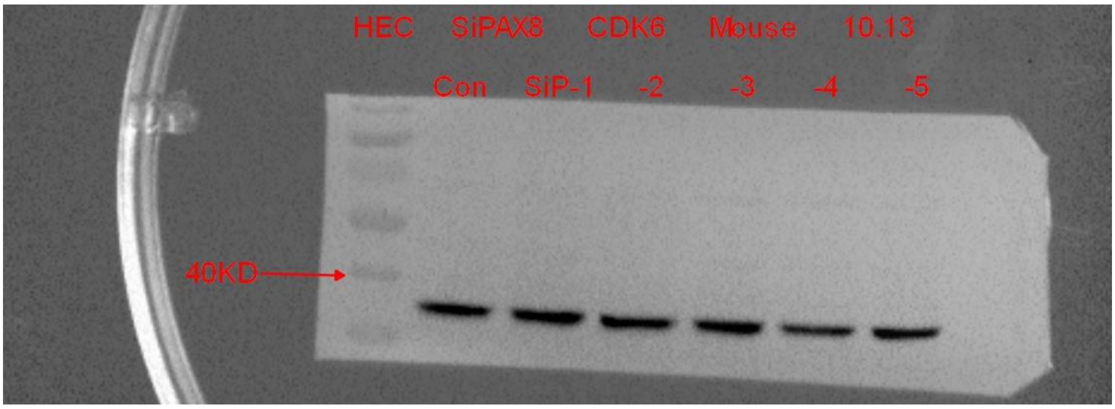

CDK6

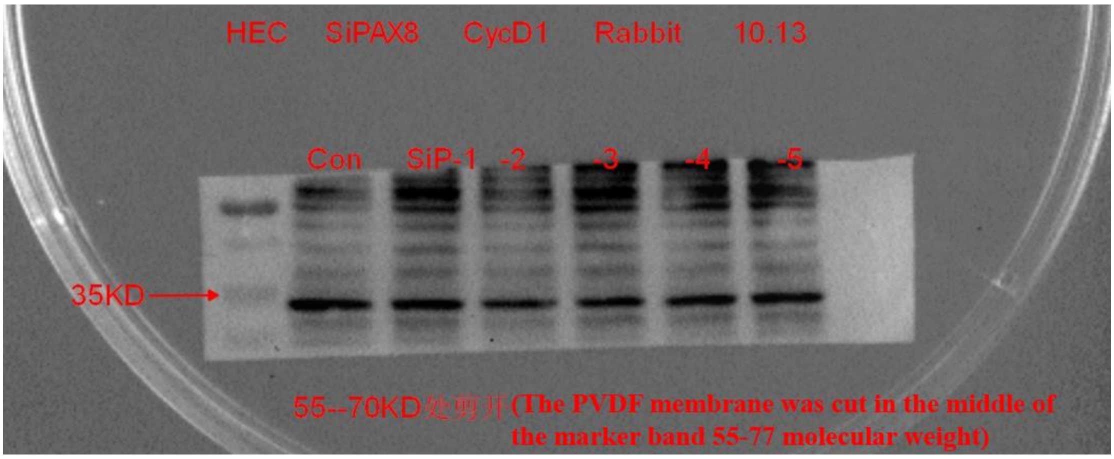

CycD1

Original western blotting data

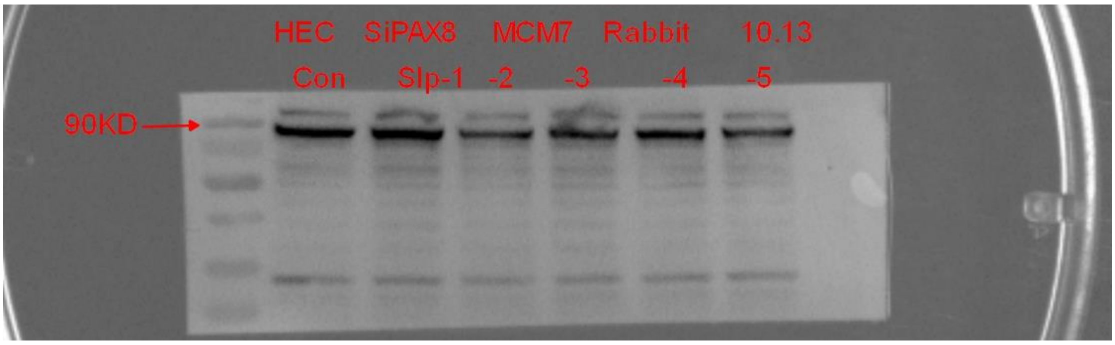

MCM7

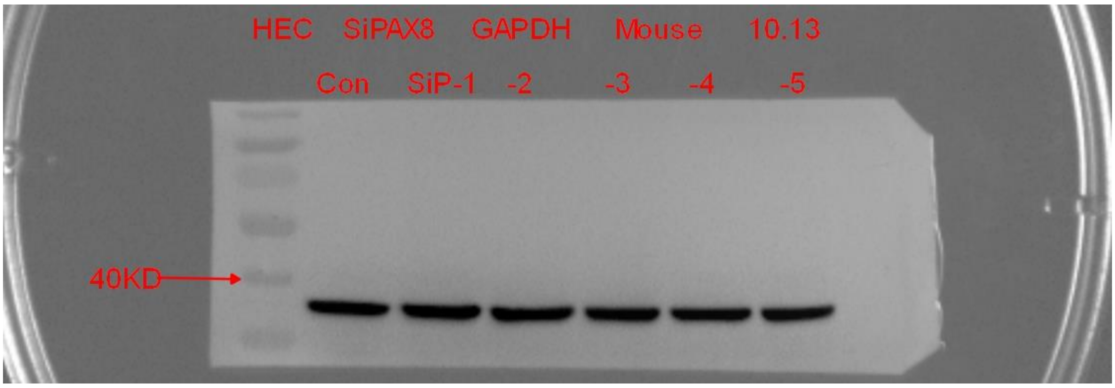

GAPDH

*Original western blotting data(Figure4 C).

Original western blotting data

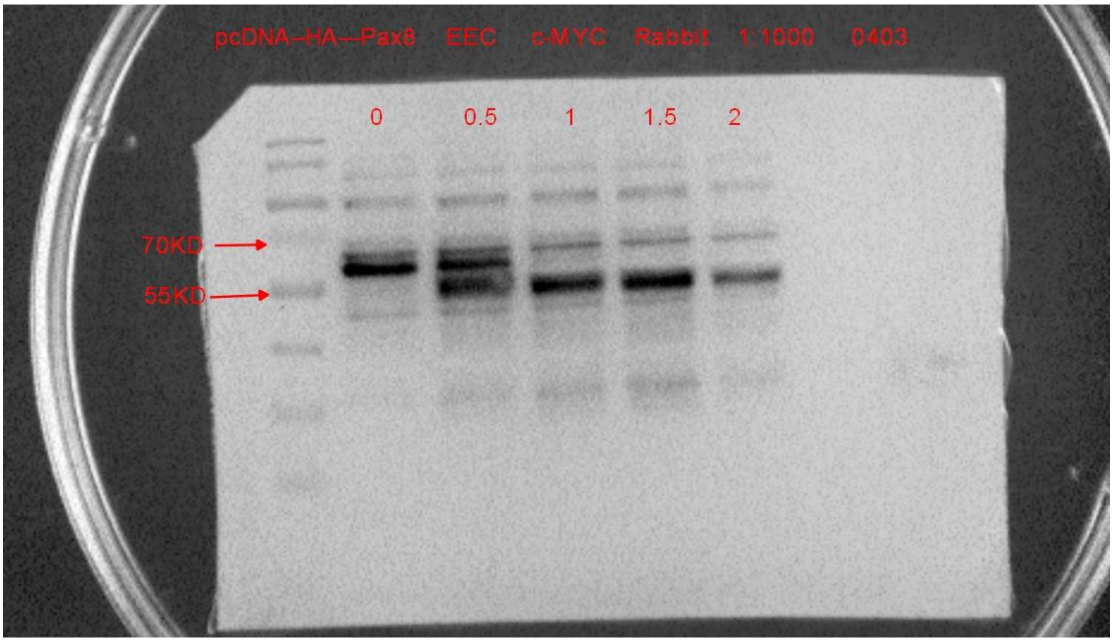

c-MYC

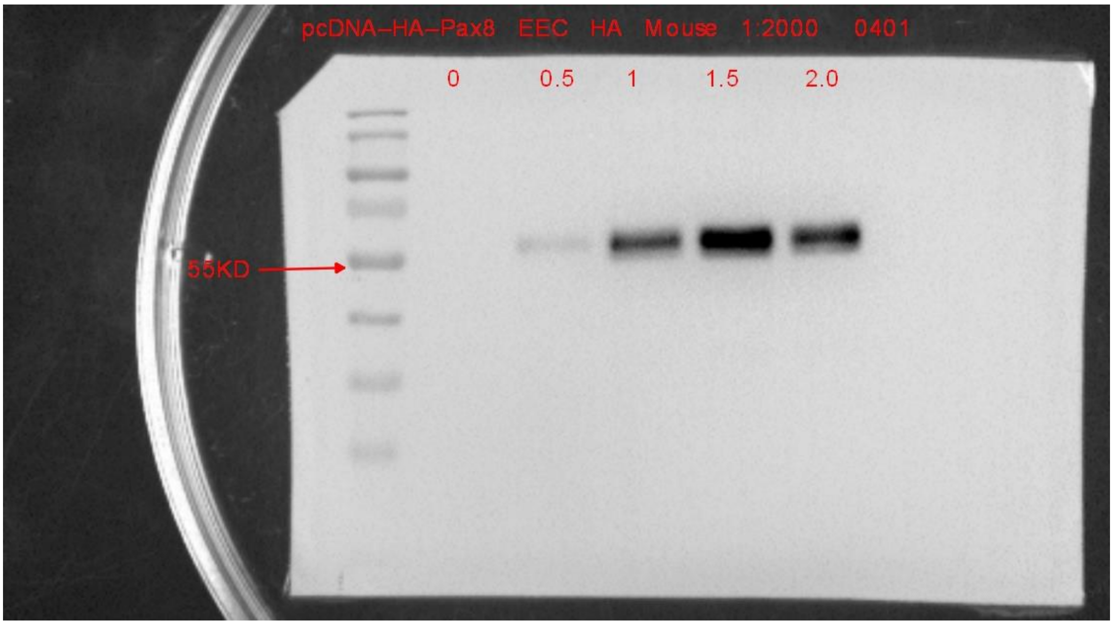

HA

Original western blotting data

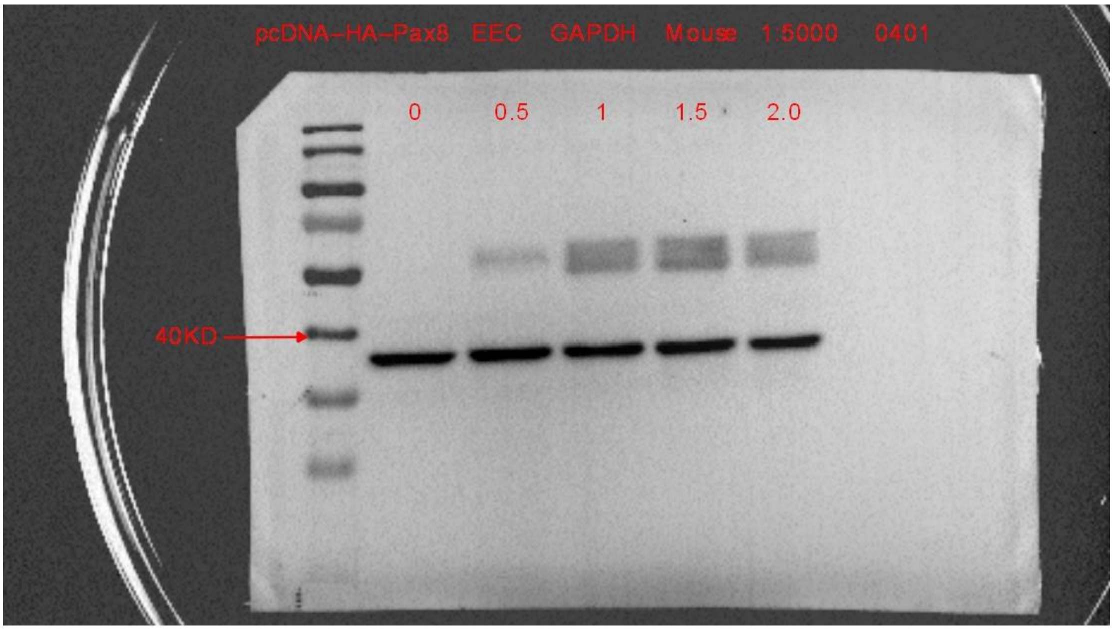

GAPDH

*Original western blotting data(Figure4 D).

Original western blotting data

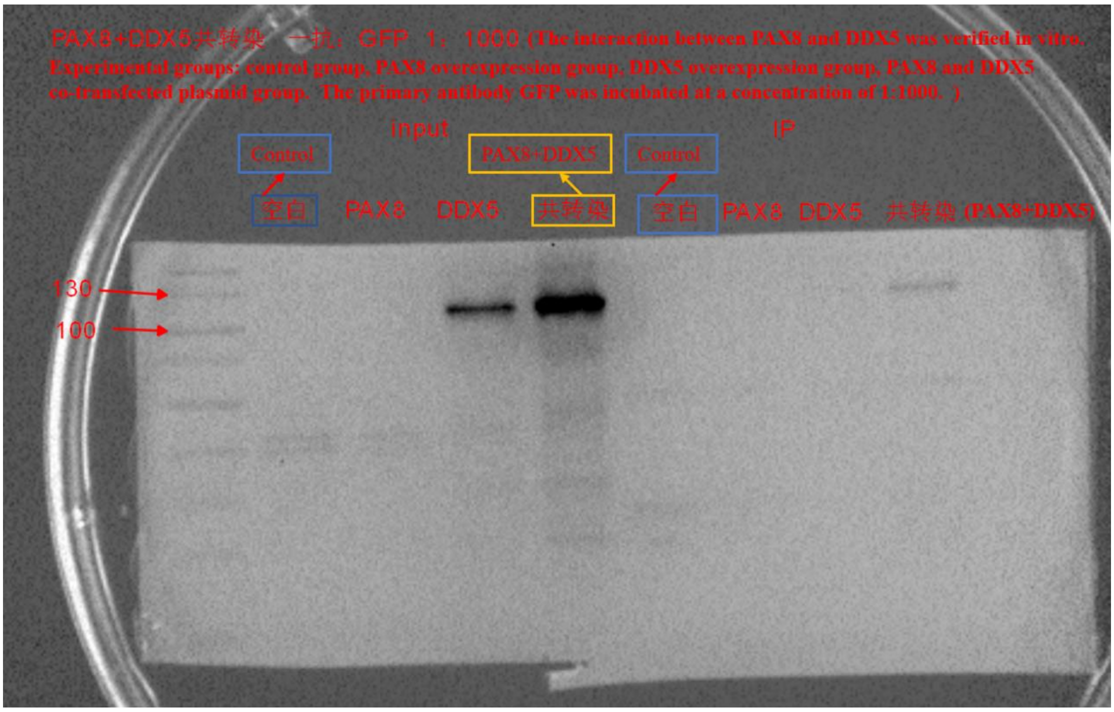

GFP

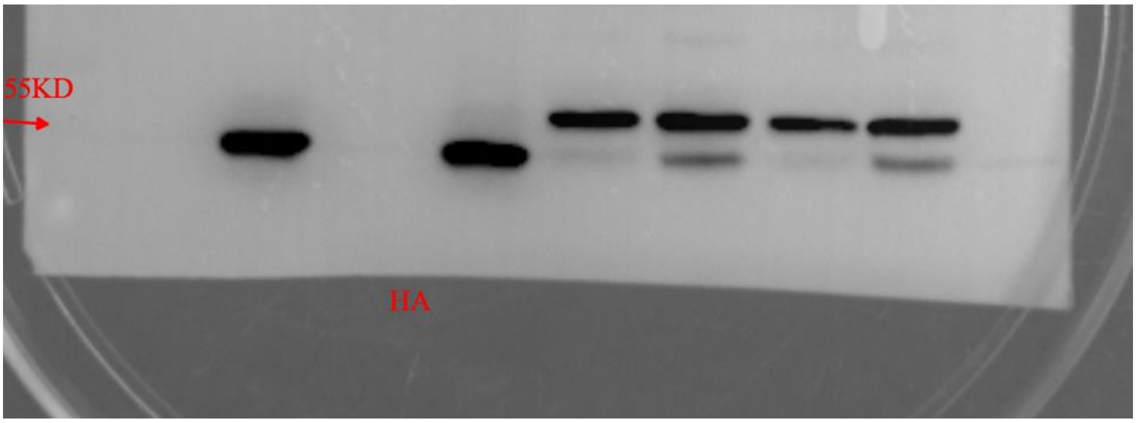

HA

*Original western blotting data(Figure5 B).

Original western blotting data

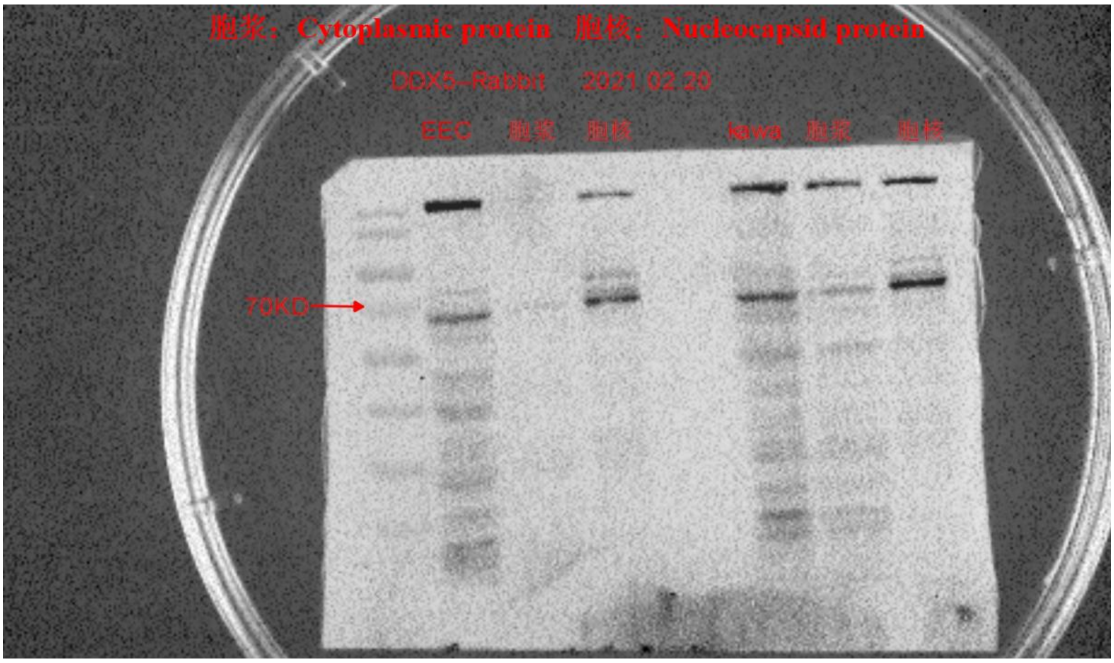

DDX5

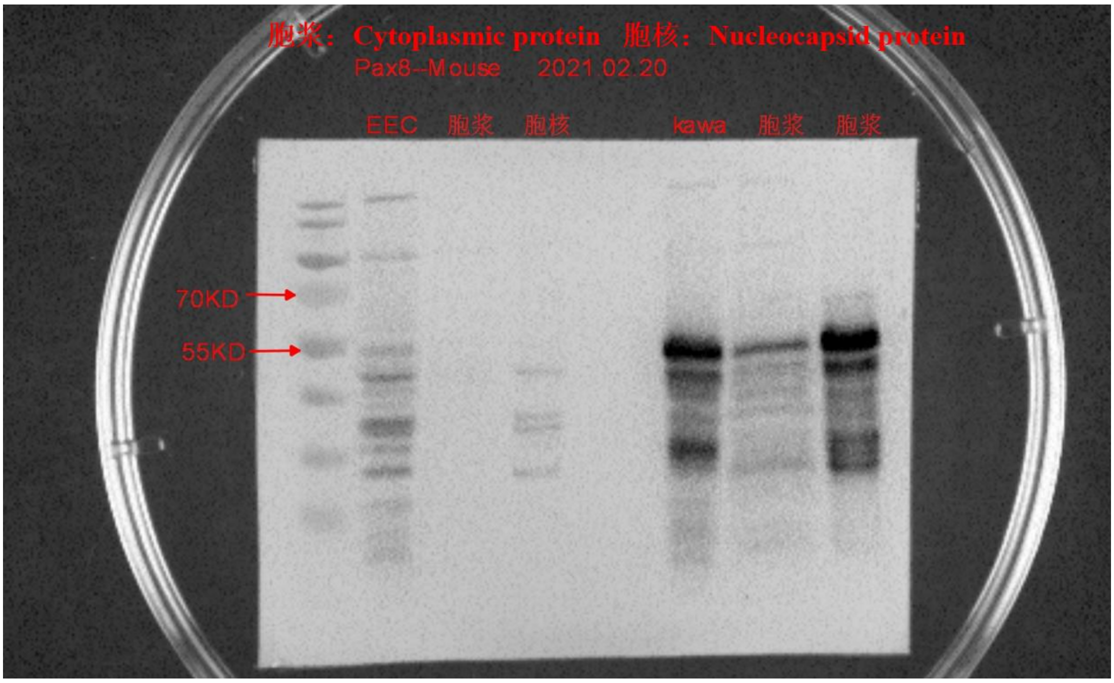

PAX8

Original western blotting data

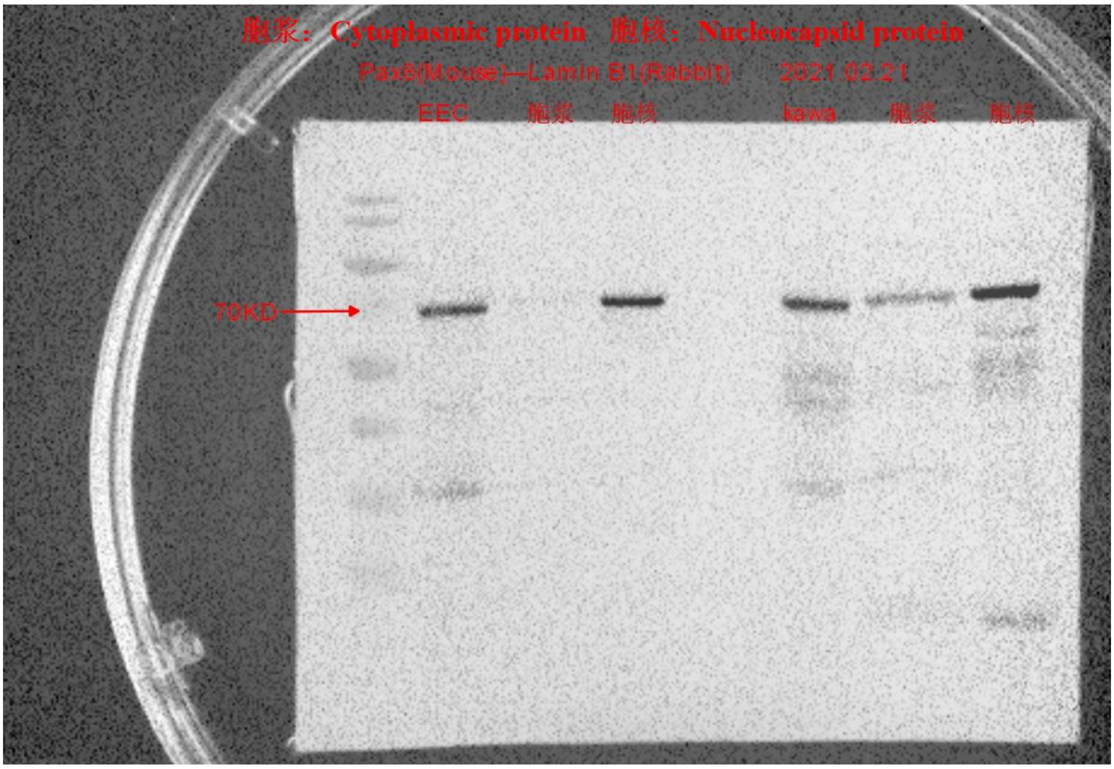

Lamin B1

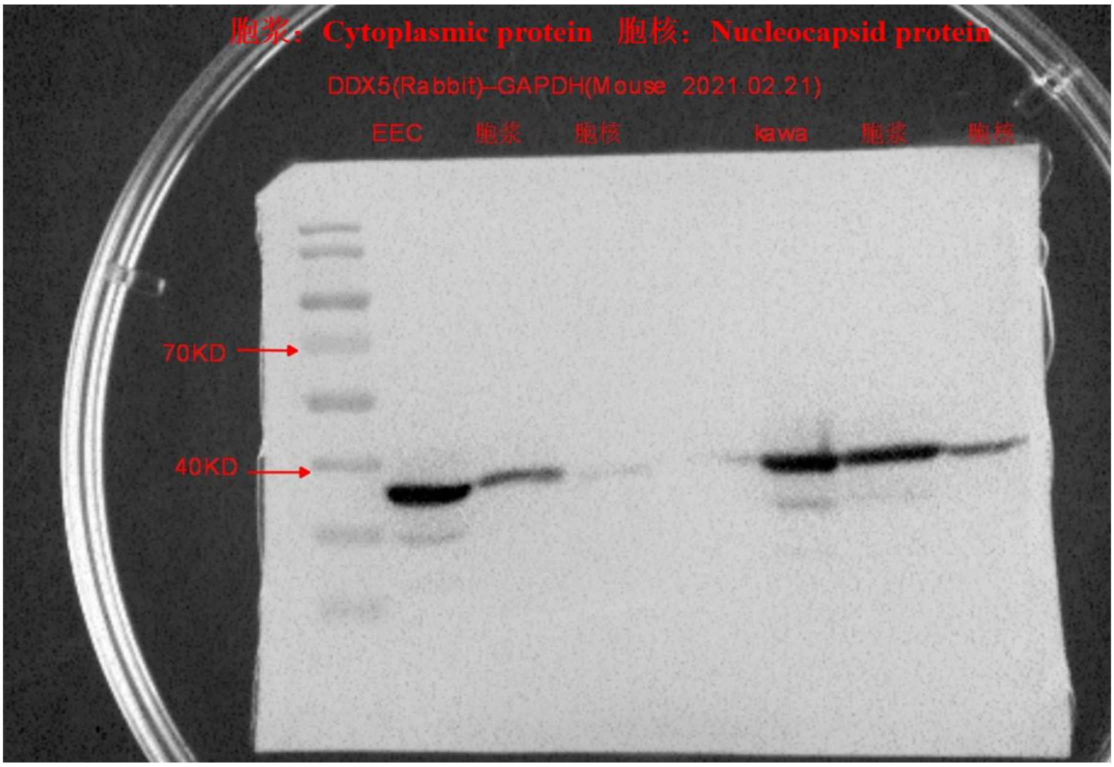

GAPDH

*Original western blotting data(Figure5 C).

Original western blotting data

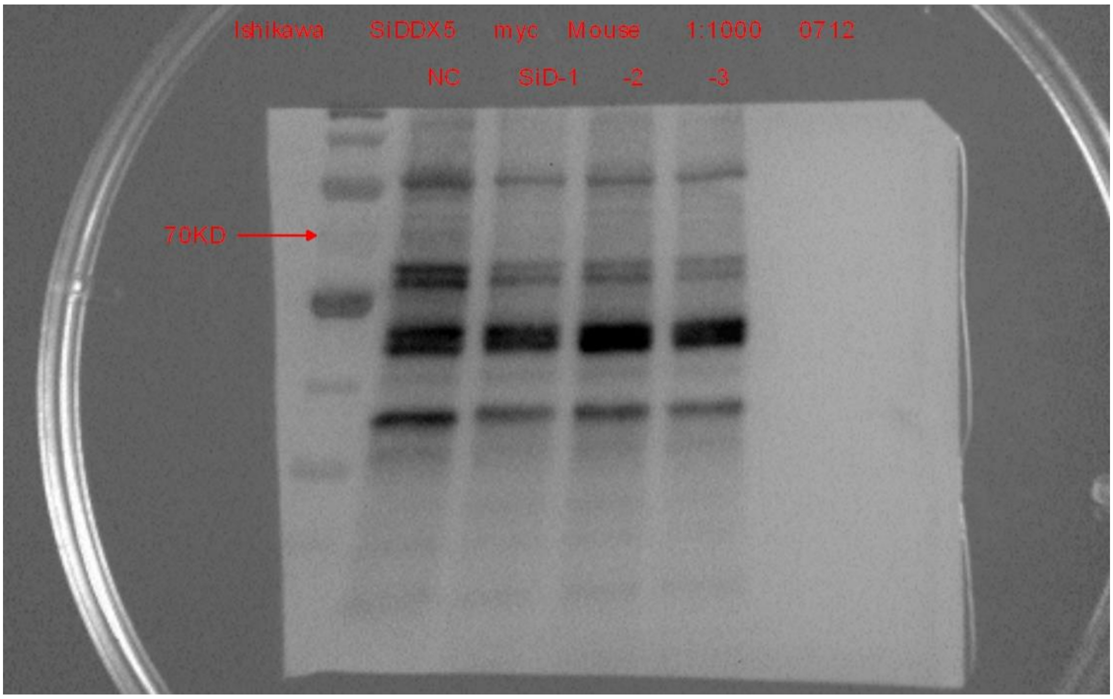

c-MYC

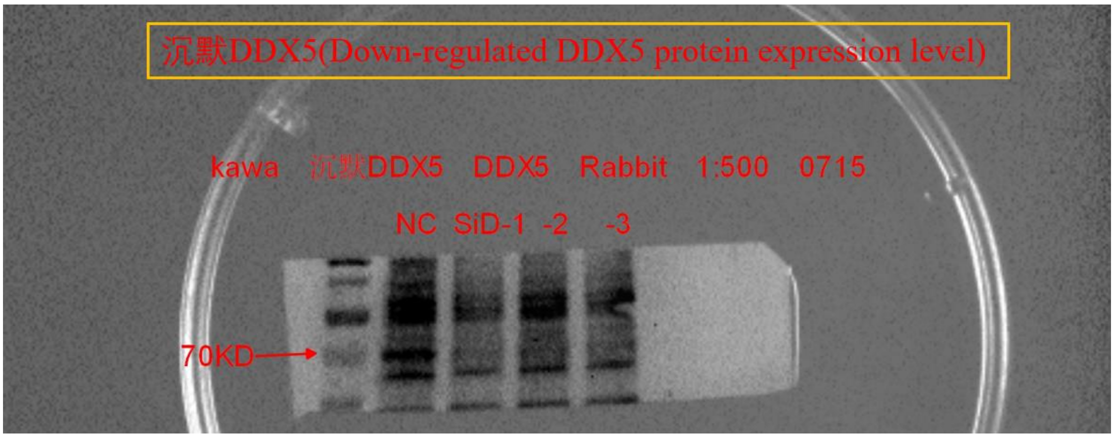

DDX5

Original western blotting data

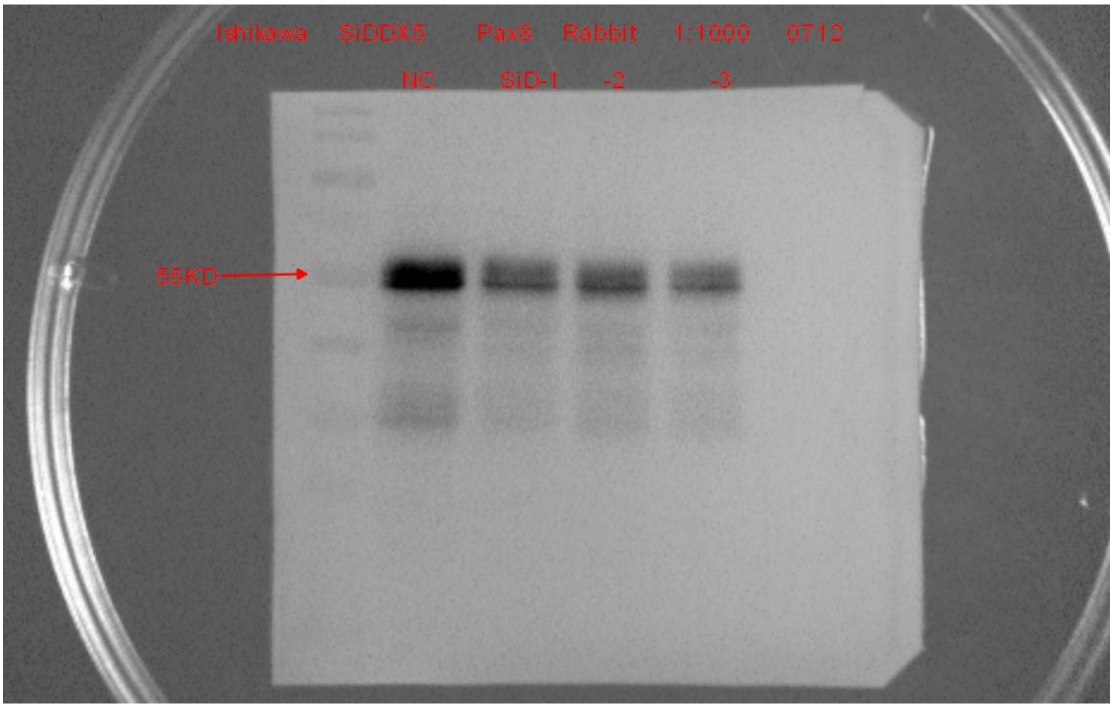

PAX8

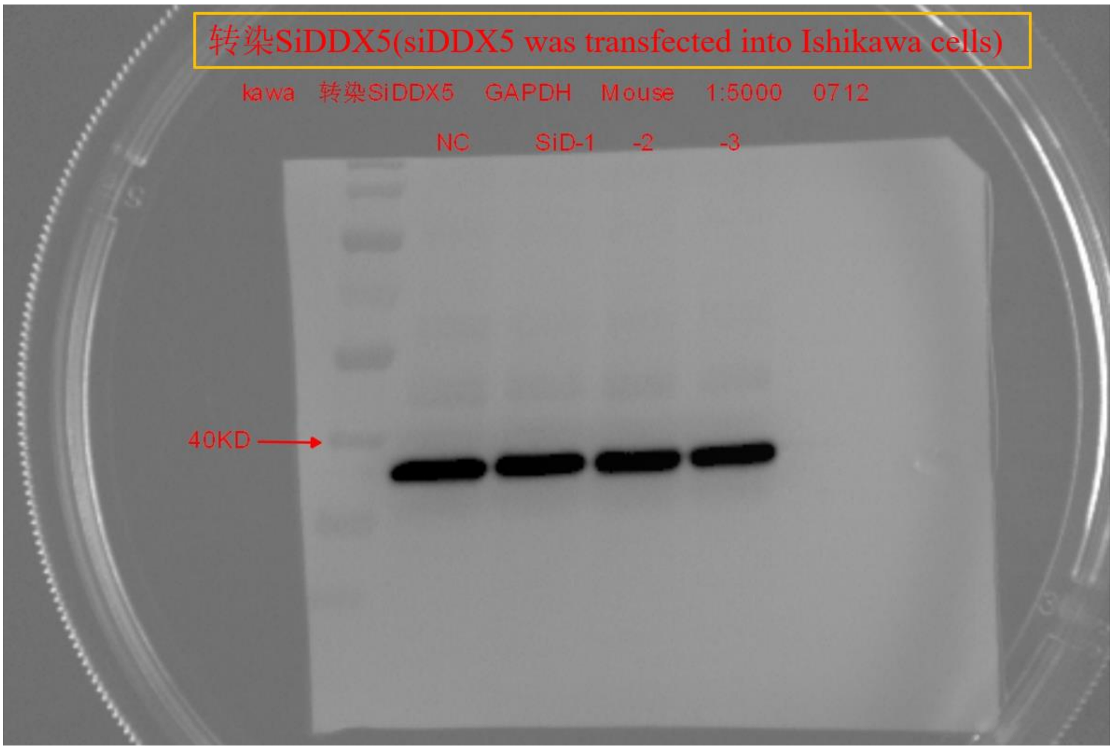

GAPDH

*Original western blotting data(Figure5 E).

Original western blotting data

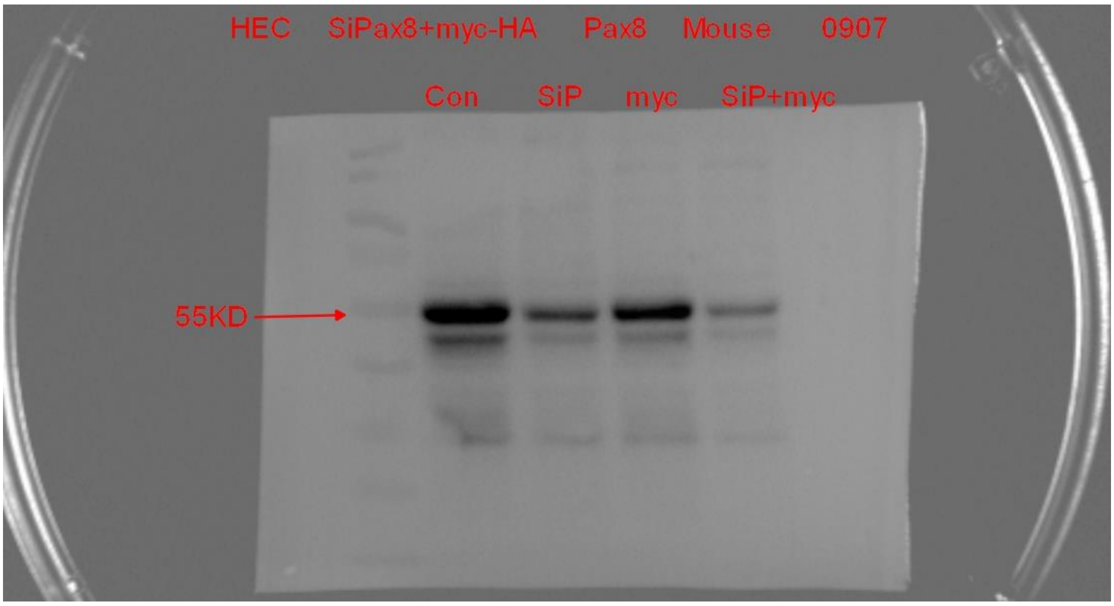

PAX8

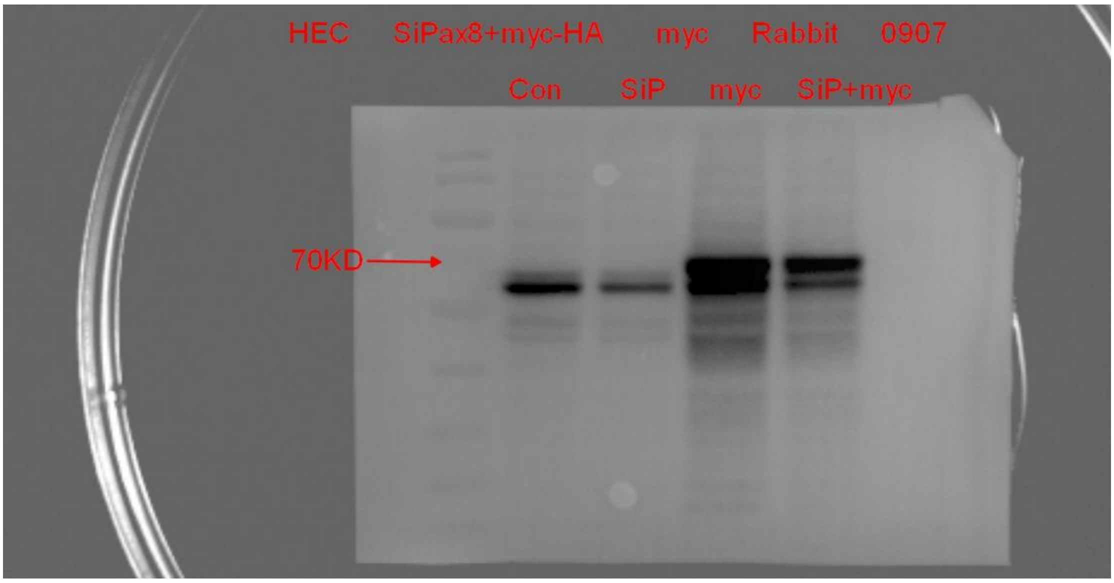

c-MYC

Original western blotting data

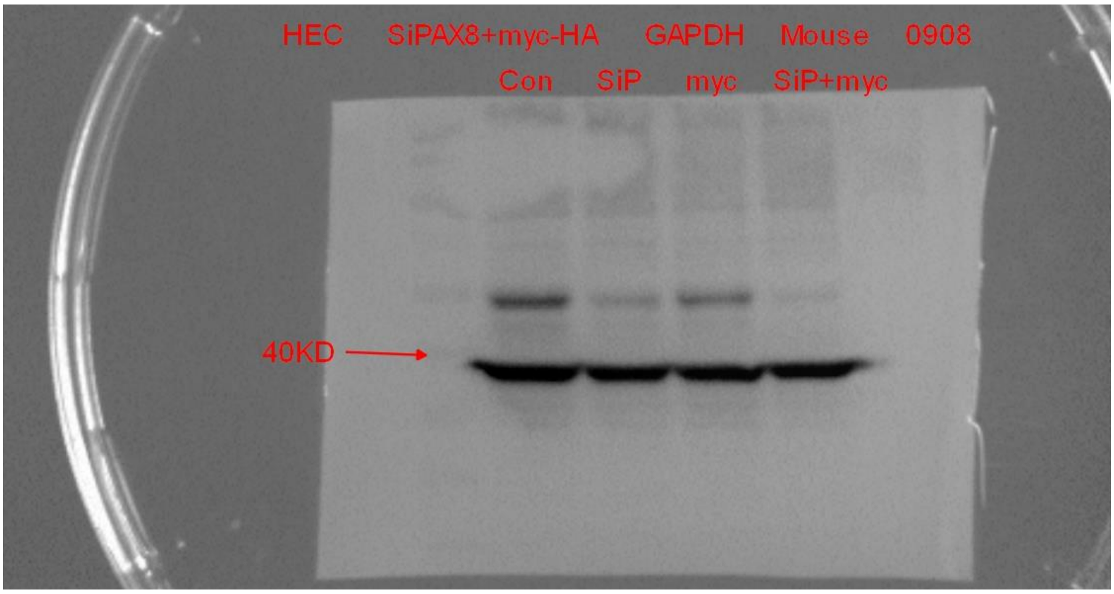

GAPDH

*Original western blotting data(Figure6 C).

Original western blotting data

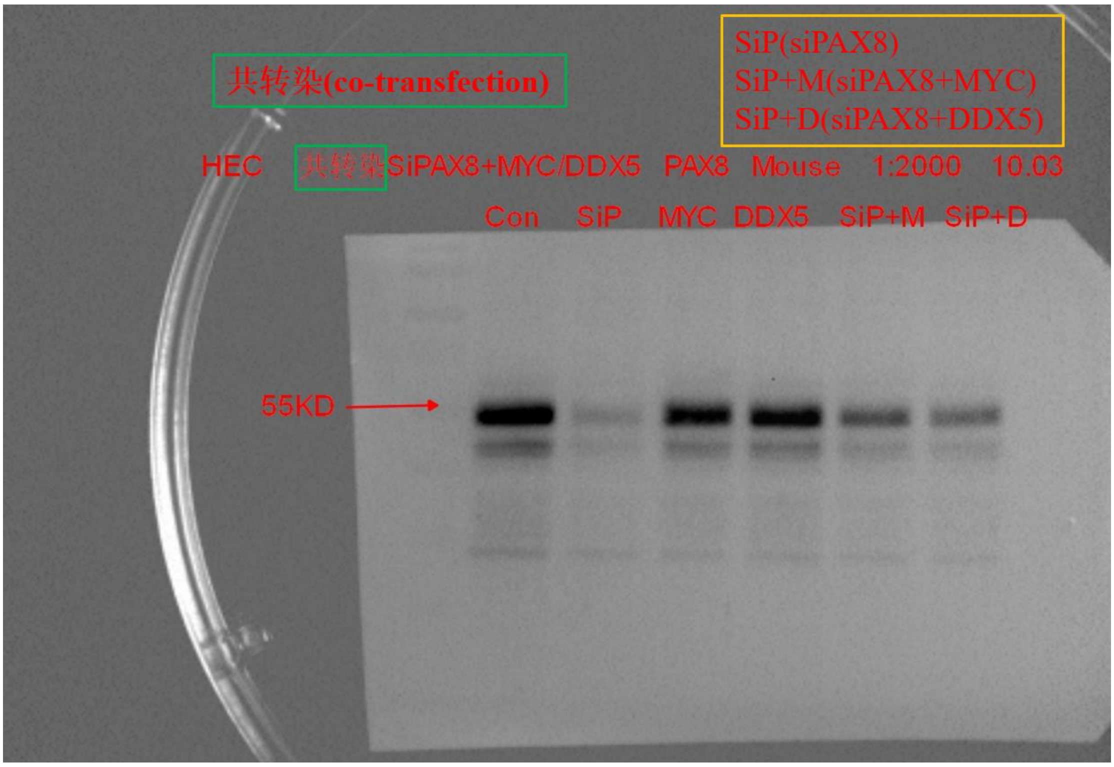

PAX8

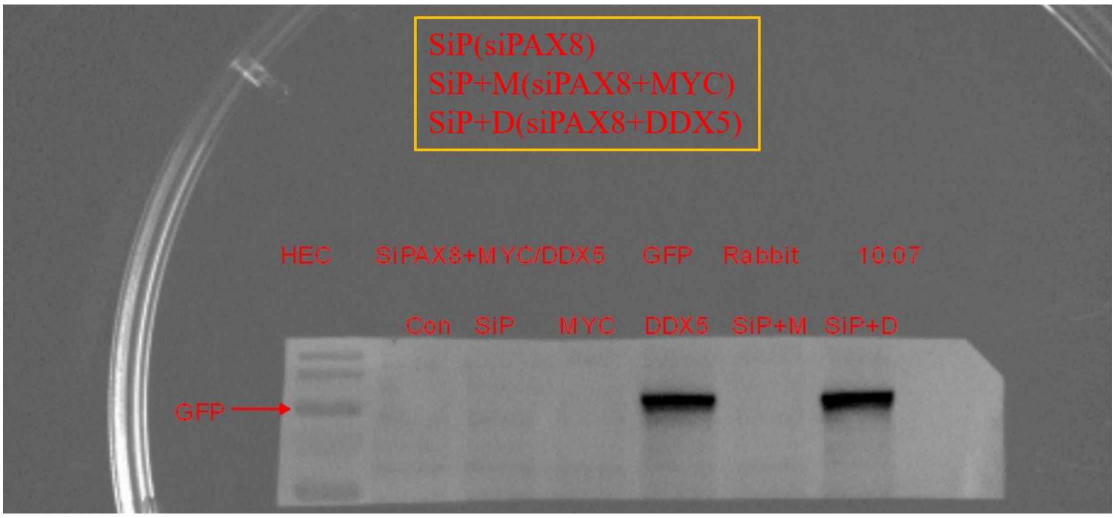

GFP

Original western blotting data

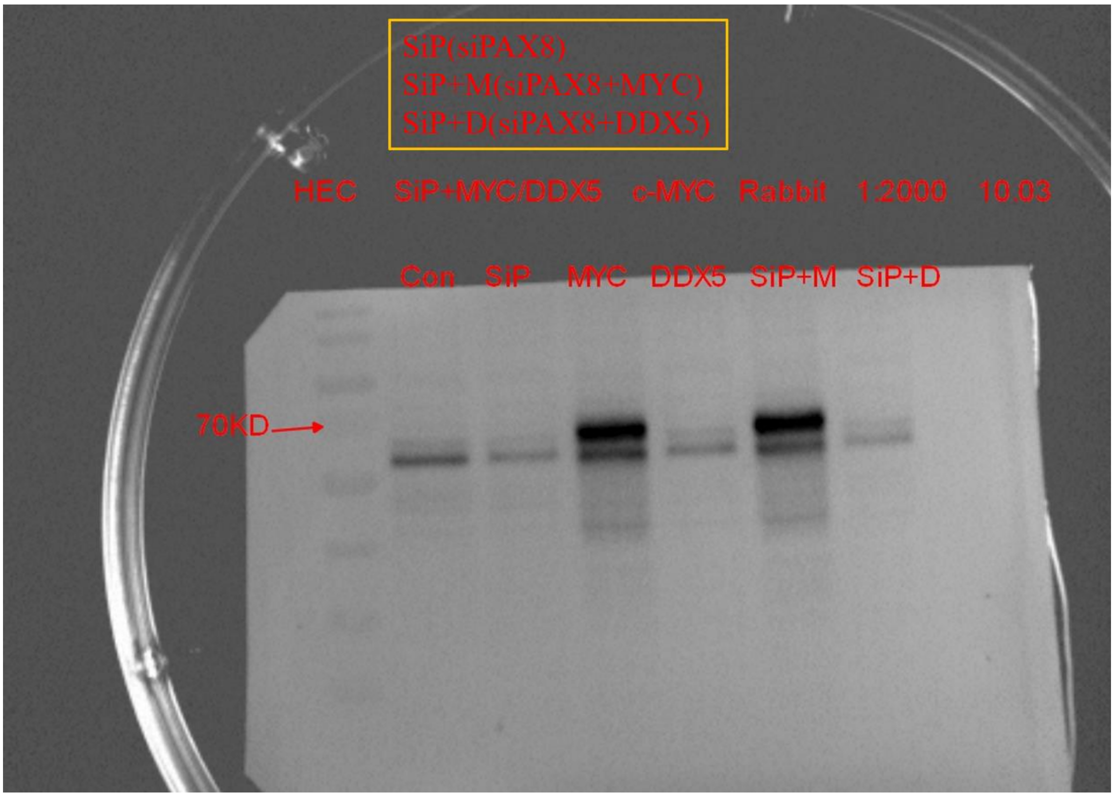

c-MYC

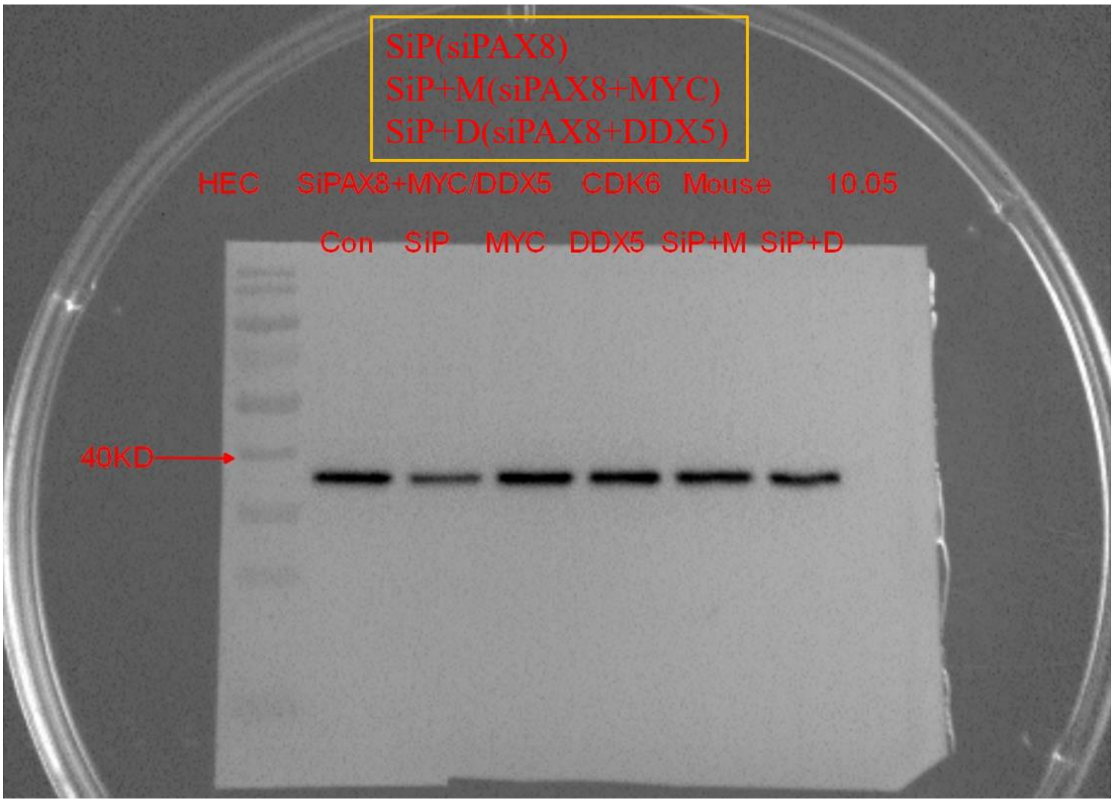

CDK6

Original western blotting data

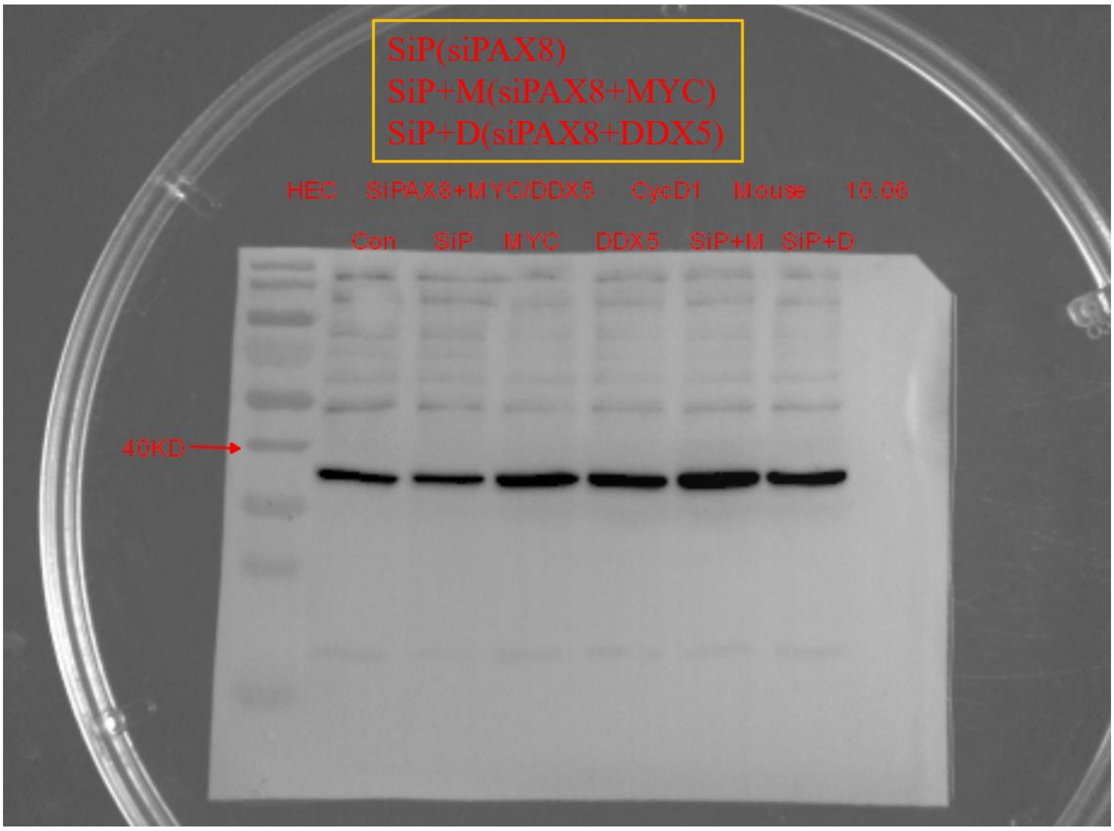

CycD1

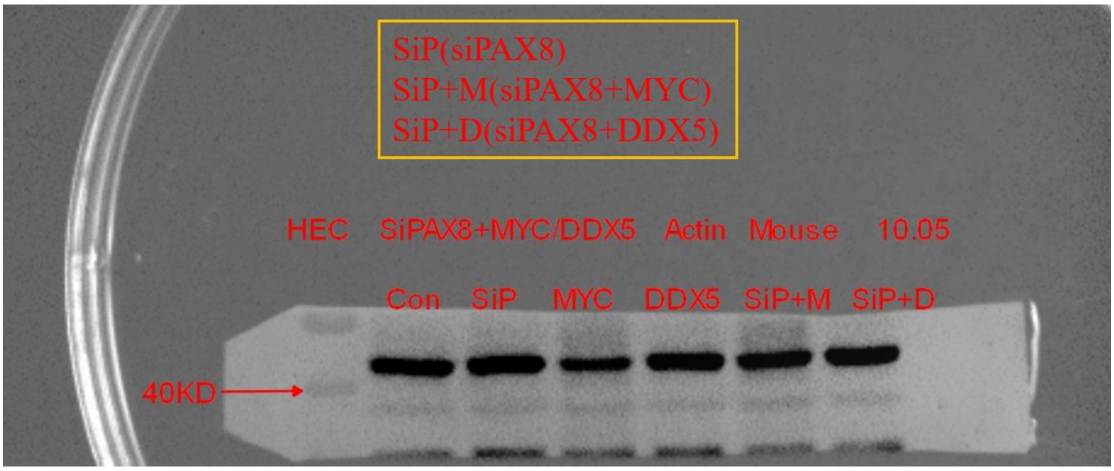

Actin

Original western blotting data

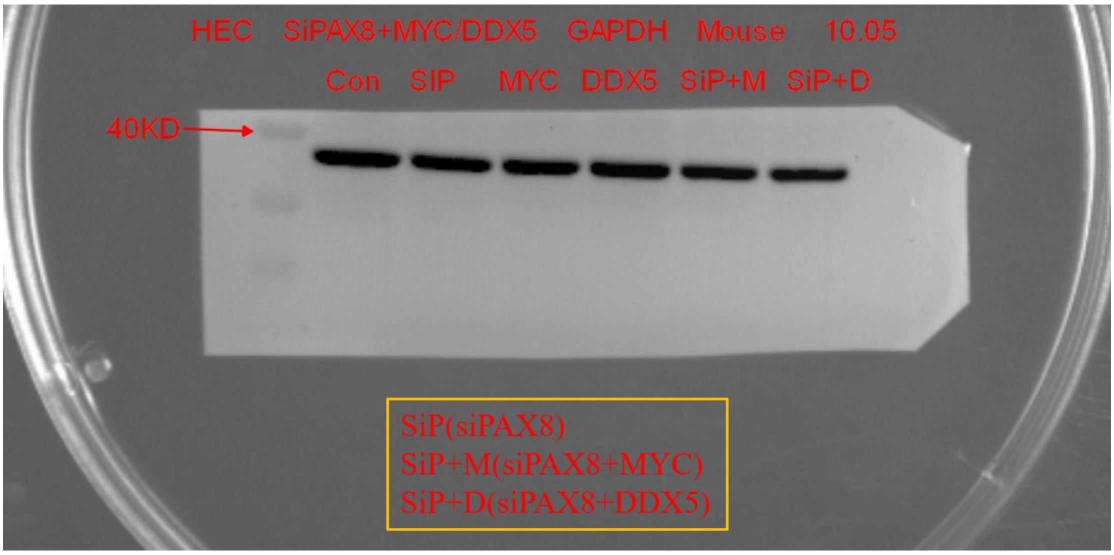

GAPDH

*Original western blotting data(Figure6 D).

Original western blotting data

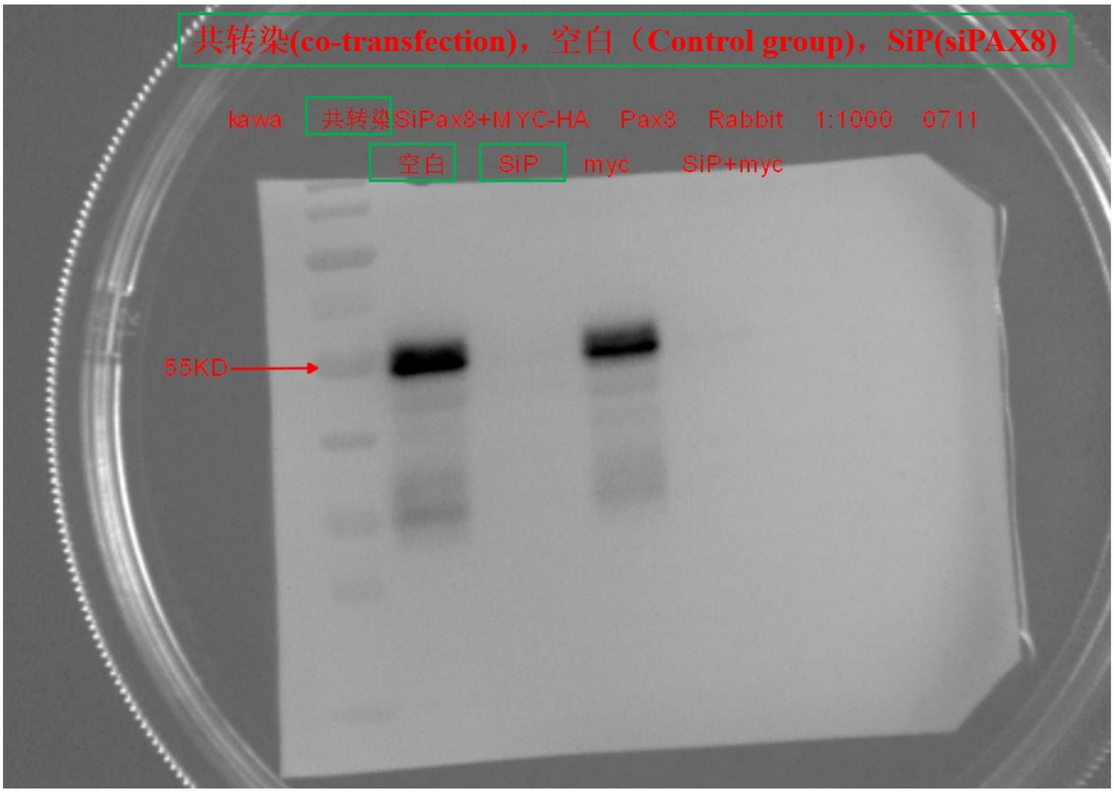

PAX8

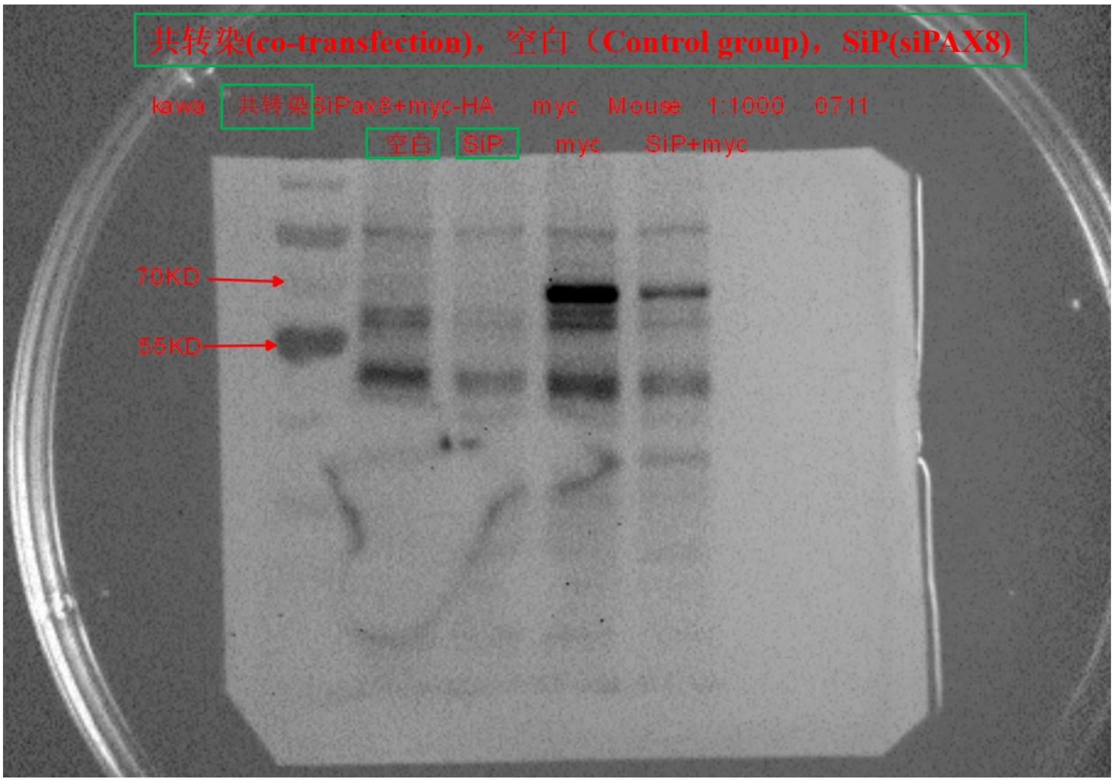

c-MYC

Original western blotting data

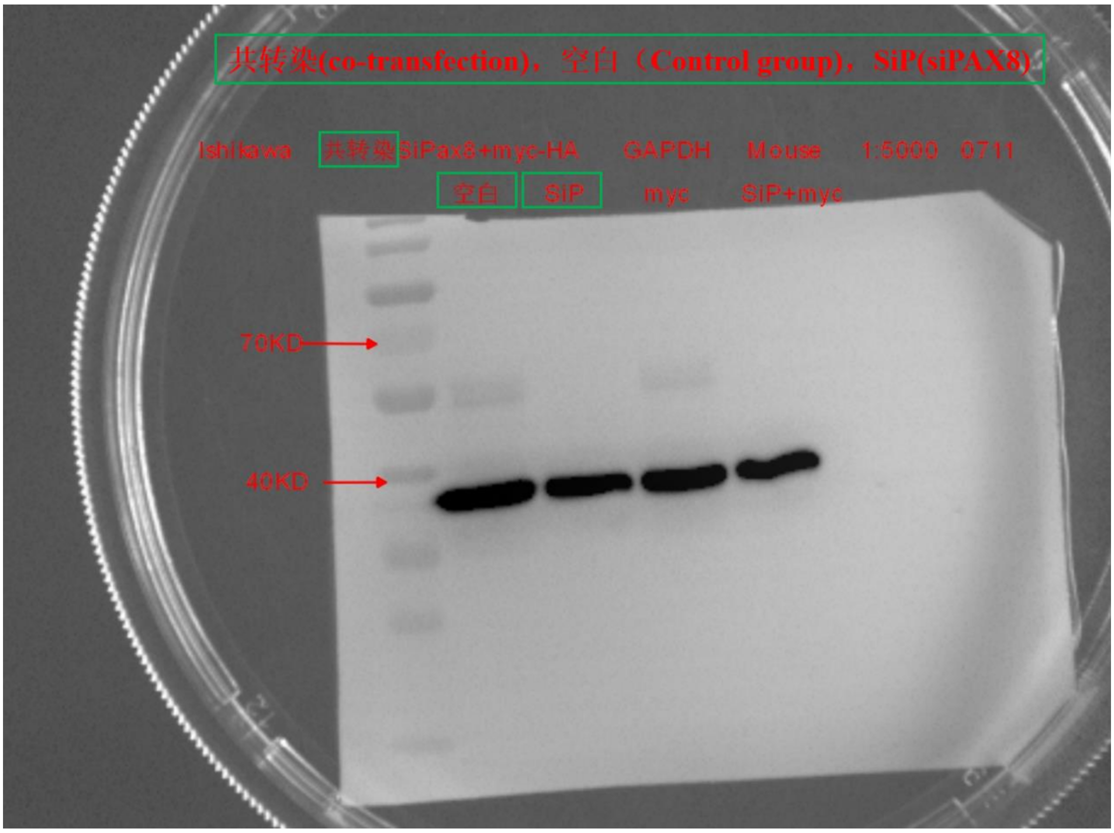

GAPDH

*Original western blotting data(Supplementary figure 5).
