# Supplementary material for: Paired box 8 facilitates the c-MYC related cell cycle progress in TP53-mutation uterine corpus endometrial carcinoma through interaction with DDX5
Source: Cell Death Discov. 2022 Jun 7;8:276. doi: 10.1038/s41420-022-01072-8 (PMC9174161; doi:10.1038/s41420-022-01072-8)
Supplement: Supplementary file 2 — SUPPLEMENTAL MATERIAL [file 41420_2022_1072_MOESM2_ESM.docx]

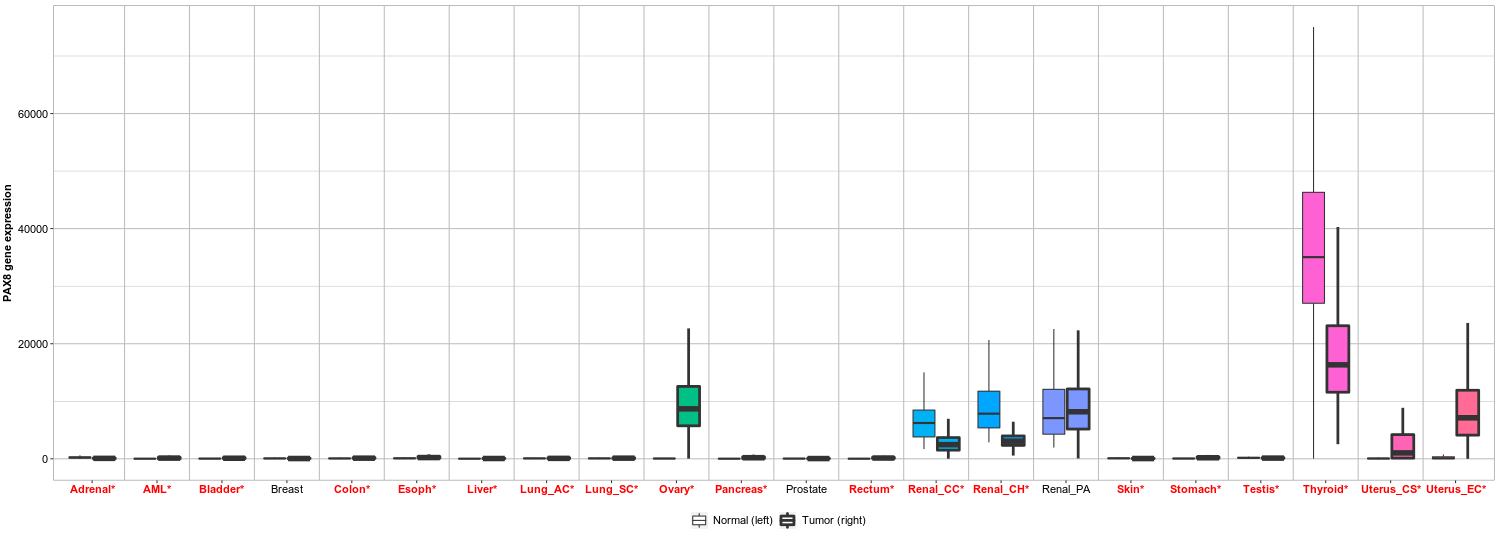


sFig.1 PAX8 mRNA expression in 22 different types of cancer as determined by normal tissue vs Cancer tissue based on TNMplot (<https://tnmplot.com>). Siginificant differences by Mann-Whitney U test are marked with red*. In case of low expression values they suggest to directly compare tumor to normal.


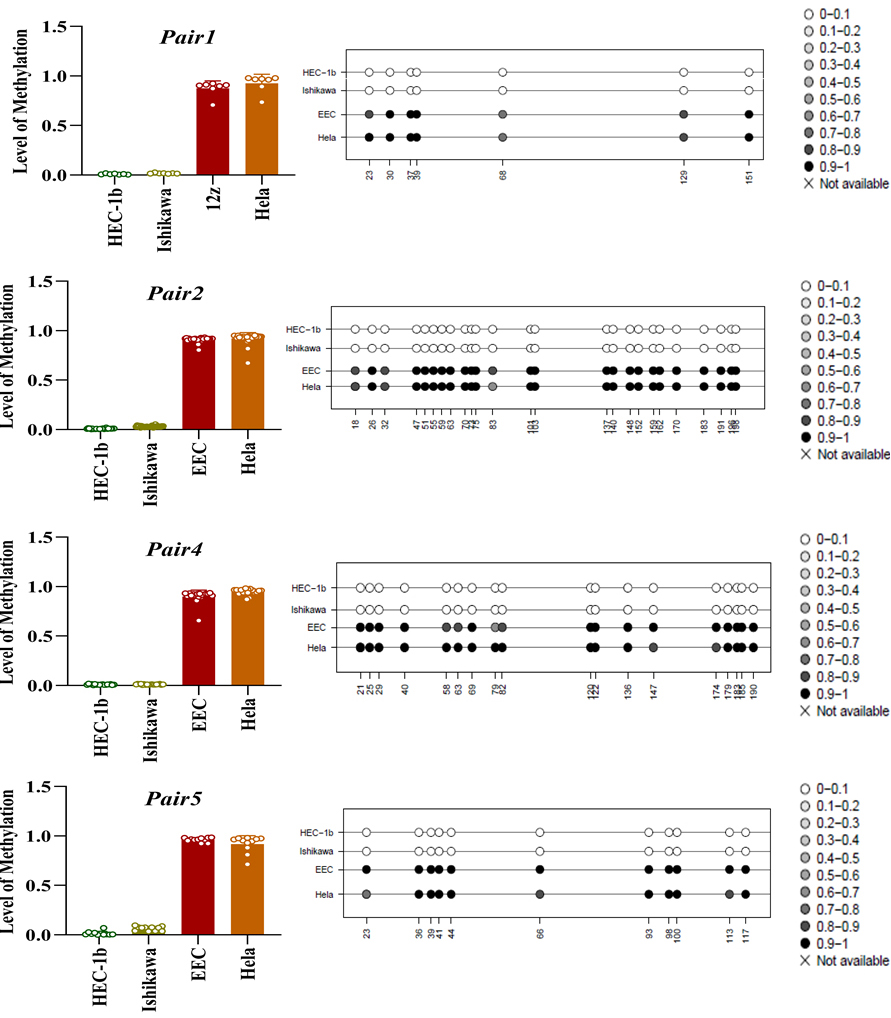


sFig.2 The methylation levels of PAX8 (pair1, 2, 4 and 5) in HEC-1B, Ishikawa, 12Z and HeLa cells were detected by bisulfite conversion sequencing. Schematic diagram of methylation information of all sites in pair1, 2, 4 and 5. Statistical table of methylation information for all sites in these pairs.


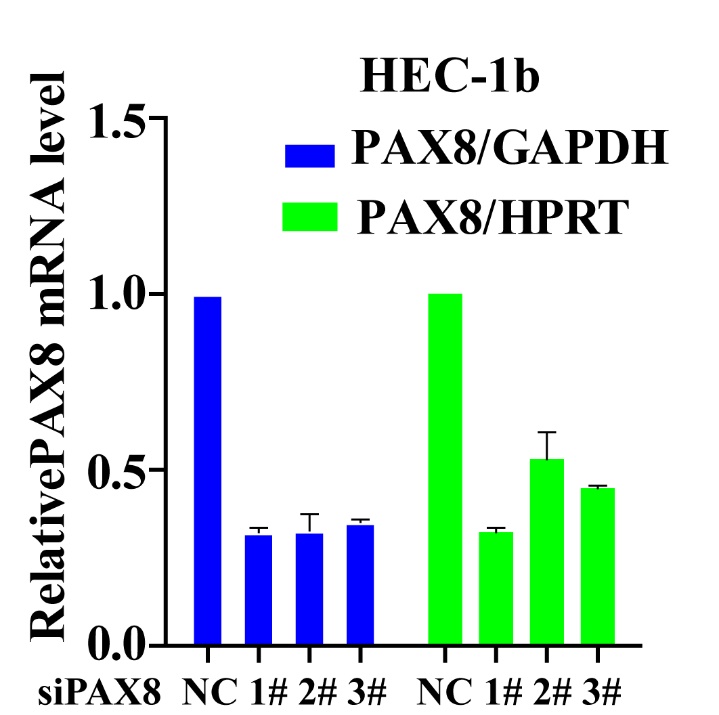


sFig.3 HEC-1B cell was transfected with PAX8 siRNA for 24 hours, RNA isolated, and RNA-Seq performed. The expression level of PAX8 mRNA after knockdown PAX8 was detected by qPCR. GAPDH and HPRT were used as a housekeeping gene.


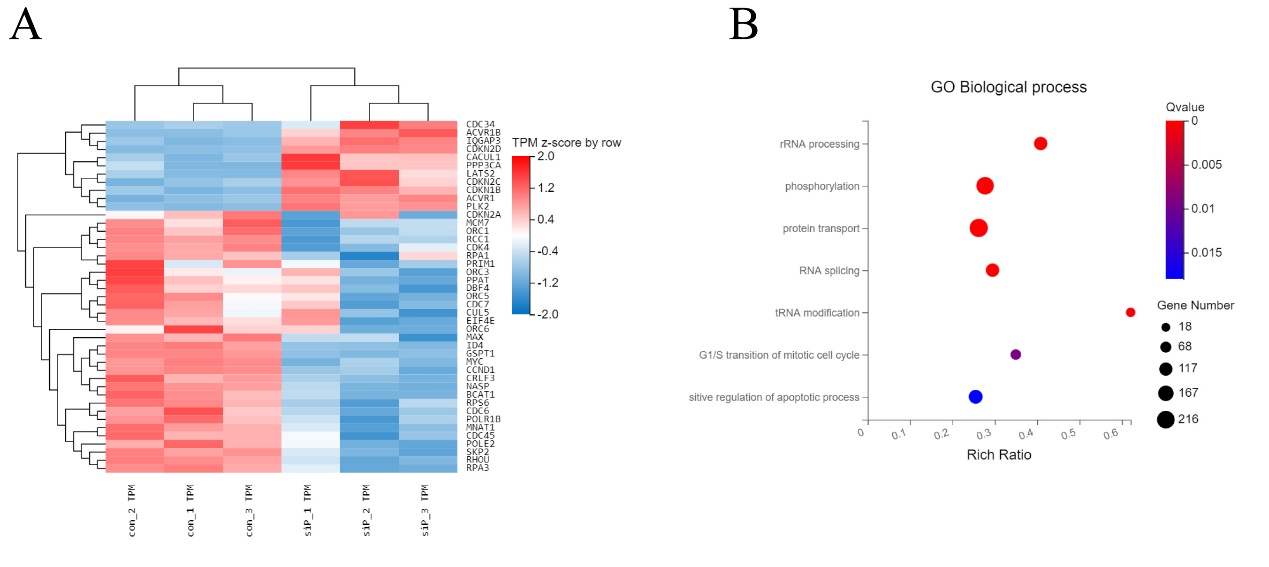


sFig.4 Heatmap showing the log2FC of significantly (FDR < 0.05) changed transcripts that are altered in expression in G1/S transition of mitotic cell cycle. Scale of heatmap is limited to [-2,2]. Gene clusters in DEGs were subject to GO enrichment analysis, and the top seven categories are shown for the correlative clusters.


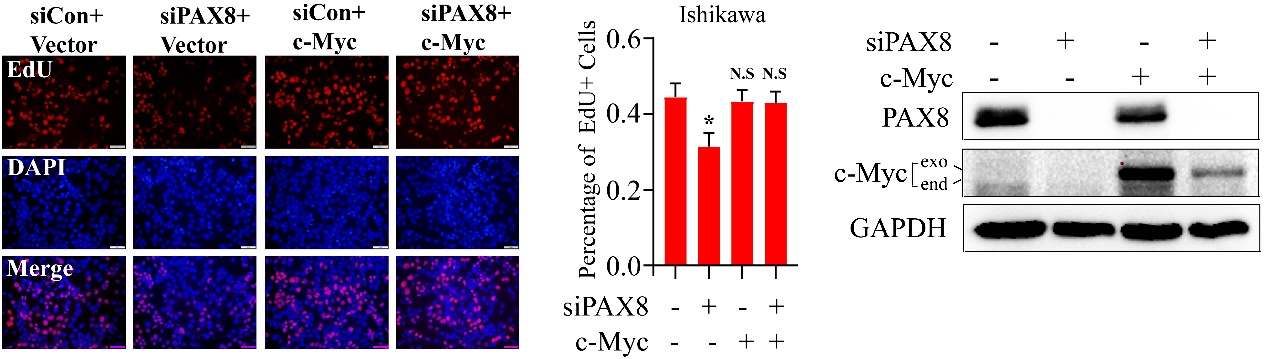


sFig.5 EdU incorporation assays of Ishikawa cell transiently transfected with siNC or siPAX8 or c-MYC or siPAX8+c-MYC. The percentage of EdU positive cells was blindly calculated with counting nine nonoverlaping fields. Values are means±s.d. Ishikawa cells were transfected with siRNAs or plasmid to knockdown PAX8 or overexpression c-MYC and DDX5, respectively. The proteins were extracted from cells for Western blotting analysis.

| RT_PAX8-F' | ACTACAAACGCCAGAACCC |
| --- | --- |
| RT_PAX8-R' | AGGGAGGTTGAATGGTTGC |
| RT_LAMP1-F' | GCTCTTCCAGTTCGGGATG |
| RT_LAMP1-R' | TAGGAATTGCCGACTGTGG |
| RT_LAMP3-F' | TCAACATCGACCCCAACG |
| RT_LAMP3-R' | AAATAGGCTCCCACTTCACTG |
| RT_RIN3-F' | CATCAAAACATGCCCGGTG |
| RT_RIN3-R' | GAGAAGGAAAGTGGACACAGAG |
| RT_NPC2-F‘ | TCTTACAGCGTCAATGTCACC |
| RT_NPC2-R‘ | TTCCACTCTTACAACCATCAGG |
| RT_MRPL3-F' | GGTAAAGGTTTTCAAGGTGTCATG |
| RT_MRPL3-R' | TTTTAGTTCCAGGCCAGACTC |
| RT_MRPS6-F' | GGAGCAATAGTGAGGGACTTG |
| RT_MRPS6-R’ | CACCATGCTTTCAACAGCTG |
| RT_MRPL20-F' | GGTCAGAACCGTGATTCGAG |
| RT_MRPL20-R' | TTAACTAAATTCCCAATGAGCGC |
| RT_MRPS21-F' | GGTCATGGCAAAACATCTGAAG |
| RT_MRPS21-R' | CCGATGCTTAATGTCCTCAATG |
| RT_FBL-F' | GTCTATGCAGTCGAGTTCTCC |
| RT_FBL-R' | TCAGCAAAGATCACATCCACC |
| RT_NOL6-F' | GTATAGACCCGGCTAGAATCTG |
| RT_NOL6-R' | CTTCCCCTCTTTGCCTGTG |
| RT_NOB1-F' | TGCACTCACATACCAGTTGG |
| RT_NOB1-R' | TTGTAGGGCAGATGGAAACC |
| RT_IMP3-F' | ACTCGTCCAAGATCAAGCG |
| RT_IMP3-R' | CCTCATAGAATCTCCAGCATCAG |
| RT_MYC-F' | TTCGGGTAGTGGAAAACCAG |
| RT_MYC-R' | AGTAGAAATACGGCTGCACC |
| RT_PCNA-F' | GTCTCTTTGGTGCAGCTCA |
| RT_PCNA-R' | ATCTTCGGCCCTTAGTGTAATG |
| RT_CDK4-F' | TTCCCATCAGCACAGTTCG |
| RT_CDK4-R' | TCTACATGCTCAAACACCAGG |
| RT_MCM7-F' | GATGCCACCTATACTTCTGCC |
| RT_MCM7-R' | TCCTTTGACATCTCCATTAGCC |

Supplementary Table1. The sequence of primers for qPCR

Supplementary Table2. IP_PAX8_Mass_Information

| **Accession** | **Gene names** | **MW [kDa]** | **Protein score** | **Sequence coverage (%)** | **# Unique Peptides** | **# Peptides** | **# PSMs** |
| --- | --- | --- | --- | --- | --- | --- | --- |
| P35527 | KRT9 | 62.03 | 1202.23 | 64.53 | 26 | 27 | 39 |
| P04264 | KRT1 | 66.00 | 885.49 | 42.70 | 19 | 23 | 29 |
| P05787 | KRT8 | 53.67 | 790.84 | 41.41 | 16 | 19 | 28 |
| P13645 | KRT10 | 58.79 | 699.84 | 39.04 | 21 | 24 | 26 |
| P35908 | KRT2 | 65.39 | 689.06 | 43.04 | 15 | 20 | 23 |
| P05783 | KRT18 | 48.03 | 503.73 | 46.74 | 15 | 17 | 19 |
| Q06710 | PAX8 | 48.19 | 453.77 | 38.22 | 14 | 14 | 17 |
| P08727 | KRT19 | 44.08 | 421.30 | 41.00 | 11 | 16 | 17 |
| Q86YZ3 | HRNR | 282.23 | 416.24 | 12.32 | 8 | 8 | 11 |
| P06748 | NPM1 | 32.55 | 393.37 | 27.55 | 6 | 6 | 9 |
| O60814 | H2BC12 | 13.88 | 339.90 | 46.83 | 1 | 6 | 14 |
| P06899 | H2BC11 | 13.90 | 339.85 | 47.62 | 1 | 6 | 14 |
| P22626 | HNRNPA2B1 | 37.41 | 264.04 | 32.01 | 7 | 9 | 12 |
| Q9BQE3 | TUBA1C | 49.86 | 249.27 | 20.71 | 7 | 7 | 8 |
| Q00839 | HNRNPU | 90.53 | 246.56 | 11.15 | 7 | 7 | 7 |
| P60709 | ACTB | 41.71 | 245.90 | 39.73 | 4 | 12 | 17 |
| P31943 | HNRNPH1 | 49.20 | 237.70 | 15.14 | 4 | 6 | 7 |
| P52272 | HNRNPM | 77.46 | 236.25 | 17.67 | 9 | 9 | 9 |
| P35579 | MYH9 | 226.39 | 233.26 | 8.37 | 12 | 12 | 13 |
| P08779 | KRT16 | 51.24 | 226.20 | 17.55 | 3 | 9 | 10 |
| P09651 | HNRNPA1 | 38.72 | 221.61 | 15.86 | 3 | 5 | 7 |
| P68104 | EEF1A1 | 50.11 | 216.07 | 13.42 | 5 | 5 | 7 |
| P61978 | HNRNPK | 50.94 | 213.79 | 21.38 | 7 | 7 | 7 |
| P11142 | HSPA8 | 70.85 | 203.66 | 12.54 | 5 | 6 | 7 |
| P68032 | ACTC1 | 41.99 | 182.90 | 27.32 | 1 | 9 | 12 |
| P07910 | HNRNPC | 33.65 | 181.18 | 26.14 | 8 | 8 | 8 |
| P23246 | SFPQ | 76.10 | 179.51 | 19.52 | 10 | 11 | 11 |
| Q02878 | RPL6 | 32.71 | 178.25 | 23.61 | 6 | 6 | 7 |
| P02768 | ALB | 69.32 | 165.23 | 4.76 | 4 | 4 | 5 |
| P62805 | H4C1 | 11.36 | 164.01 | 31.07 | 4 | 4 | 5 |
| P36578 | RPL4 | 47.67 | 163.07 | 18.50 | 8 | 8 | 8 |
| P52597 | HNRNPF | 45.64 | 149.13 | 12.29 | 2 | 4 | 4 |
| P02538 | KRT6A | 60.01 | 147.17 | 12.41 | 1 | 7 | 8 |
| P19338 | NCL | 76.57 | 146.70 | 9.58 | 7 | 7 | 8 |
| P13647 | KRT5 | 62.34 | 140.15 | 16.10 | 4 | 10 | 11 |
| P68371 | TUBB4B | 49.80 | 139.35 | 21.35 | 6 | 6 | 6 |
| P46087 | NOP2 | 89.25 | 132.65 | 4.93 | 3 | 3 | 3 |
| Q04695 | KRT17 | 48.08 | 131.83 | 14.81 | 3 | 8 | 8 |
| Q07020 | RPL18 | 21.62 | 125.70 | 18.09 | 3 | 3 | 3 |
| P68871 | HBB | 15.99 | 121.15 | 28.57 | 3 | 3 | 3 |
| P18124 | RPL7 | 29.21 | 120.87 | 12.10 | 3 | 3 | 4 |
| Q15233 | NONO | 54.20 | 117.41 | 12.74 | 4 | 5 | 5 |
| Q13151 | HNRNPA0 | 30.82 | 116.00 | 14.10 | 2 | 3 | 4 |
| P62917 | RPL8 | 28.01 | 115.81 | 15.95 | 4 | 4 | 4 |
| P46781 | RPS9 | 22.58 | 114.24 | 25.77 | 5 | 5 | 5 |
| P17844 | DDX5 | 69.10 | 112.57 | 8.79 | 5 | 5 | 5 |
| Q13263 | TRIM28 | 88.49 | 112.22 | 4.79 | 3 | 3 | 3 |
| P61513 | RPL37A | 10.27 | 109.40 | 19.57 | 1 | 1 | 1 |
| P04406 | GAPDH | 36.03 | 108.45 | 8.36 | 2 | 2 | 2 |
| Q08211 | DHX9 | 140.87 | 108.02 | 5.59 | 6 | 6 | 6 |
| P62263 | RPS14 | 16.26 | 107.45 | 15.89 | 2 | 2 | 2 |
| P10412 | H1-4 | 21.85 | 102.01 | 21.92 | 2 | 6 | 6 |
| P16403 | H1-2 | 21.35 | 101.87 | 25.35 | 2 | 5 | 5 |
| P62277 | RPS13 | 17.21 | 101.62 | 29.14 | 4 | 4 | 4 |
| P38159 | RBMX | 42.31 | 97.23 | 5.37 | 2 | 2 | 2 |
| P13804 | ETFA | 35.06 | 97.06 | 5.71 | 1 | 1 | 2 |
| Q96KK5 | H2AC12 | 13.90 | 96.99 | 27.34 | 1 | 3 | 4 |
| P02545 | LMNA | 74.09 | 95.60 | 5.87 | 3 | 3 | 3 |
| P39023 | RPL3 | 46.08 | 94.96 | 10.67 | 3 | 3 | 3 |
| P51991 | HNRNPA3 | 39.57 | 94.88 | 7.67 | 1 | 2 | 3 |
| P11021 | HSPA5 | 72.29 | 94.02 | 7.19 | 3 | 4 | 4 |
| P38646 | HSPA9 | 73.63 | 93.08 | 3.09 | 2 | 2 | 2 |
| P0CG48 | UBC | 76.99 | 89.46 | 32.85 | 2 | 2 | 2 |
| P62241 | RPS8 | 24.19 | 88.40 | 13.46 | 2 | 2 | 2 |
| P15880 | RPS2 | 31.30 | 88.16 | 6.83 | 2 | 2 | 2 |
| P50914 | RPL14 | 23.42 | 86.33 | 5.58 | 1 | 1 | 1 |
| P62847 | RPS24 | 15.41 | 84.20 | 20.30 | 2 | 2 | 2 |
| P47914 | RPL29 | 17.74 | 83.93 | 9.43 | 1 | 1 | 1 |
| Q16629 | SRSF7 | 27.35 | 82.56 | 10.92 | 1 | 2 | 2 |
| P43243 | MATR3 | 94.56 | 82.26 | 5.08 | 3 | 3 | 3 |
| Q8TDN6 | BRIX1 | 41.37 | 81.51 | 8.78 | 2 | 2 | 2 |
| P61353 | RPL27 | 15.79 | 78.42 | 18.38 | 3 | 3 | 3 |
| P61313 | RPL15 | 24.13 | 74.63 | 10.29 | 2 | 2 | 2 |
| P26373 | RPL13 | 24.25 | 73.48 | 22.27 | 5 | 5 | 6 |
| P25705 | ATP5F1A | 59.71 | 72.29 | 3.44 | 2 | 2 | 3 |
| P81605 | DCD | 11.28 | 71.67 | 22.73 | 2 | 2 | 2 |
| O00571 | DDX3X | 73.20 | 69.91 | 1.81 | 1 | 1 | 1 |
| P23396 | RPS3 | 26.67 | 69.58 | 5.35 | 1 | 1 | 1 |
| O43143 | DHX15 | 90.88 | 69.23 | 1.51 | 1 | 1 | 1 |
| A0A075B6P5 | IGKV2-28 | 12.95 | 68.90 | 10.83 | 1 | 1 | 1 |
| P32119 | PRDX2 | 21.88 | 68.08 | 8.59 | 1 | 1 | 1 |
| P62701 | RPS4X | 29.58 | 66.88 | 16.35 | 4 | 4 | 4 |
| P83731 | RPL24 | 17.77 | 65.22 | 8.28 | 1 | 1 | 1 |
| Q92841 | DDX17 | 80.22 | 64.13 | 3.70 | 2 | 2 | 2 |
| P12956 | XRCC6 | 69.80 | 63.84 | 2.63 | 1 | 1 | 1 |
| Q07955 | SRSF1 | 27.73 | 63.26 | 9.68 | 2 | 2 | 2 |
| O76021 | RSL1D1 | 54.94 | 62.71 | 10.20 | 4 | 4 | 4 |
| P40429 | RPL13A | 23.56 | 62.22 | 12.32 | 3 | 3 | 3 |
| Q9NR30 | DDX21 | 87.29 | 61.54 | 5.75 | 4 | 4 | 4 |
| Q9Y3U8 | RPL36 | 12.25 | 60.83 | 10.48 | 1 | 1 | 1 |
| P62249 | RPS16 | 16.44 | 60.39 | 6.85 | 1 | 1 | 1 |
| P62913 | RPL11 | 20.24 | 59.51 | 5.06 | 1 | 1 | 1 |
| O76031 | CLPX | 69.18 | 59.43 | 2.05 | 1 | 1 | 1 |
| P62244 | RPS15A | 14.83 | 57.69 | 10.77 | 1 | 1 | 1 |
| P62266 | RPS23 | 15.80 | 57.34 | 7.69 | 1 | 1 | 1 |
| P46779 | RPL28 | 15.74 | 57.22 | 8.03 | 1 | 1 | 1 |
| P09429 | HMGB1 | 24.88 | 57.17 | 6.05 | 1 | 1 | 1 |
| P20700 | LMNB1 | 66.37 | 55.77 | 1.71 | 1 | 1 | 1 |
| P62826 | RAN | 24.41 | 55.21 | 9.72 | 2 | 2 | 2 |
| P62854 | RPS26 | 13.01 | 54.22 | 13.04 | 1 | 1 | 1 |
| P42167 | TMPO | 50.64 | 54.06 | 6.17 | 2 | 2 | 2 |
| P18621 | RPL17 | 21.38 | 52.58 | 7.61 | 1 | 1 | 1 |
| Q9Y6R1 | SLC4A4 | 121.38 | 52.36 | 0.65 | 1 | 1 | 1 |
| P62899 | RPL31 | 14.45 | 52.29 | 7.20 | 1 | 1 | 1 |
| P13639 | EEF2 | 95.28 | 52.12 | 1.05 | 1 | 1 | 1 |
| Q8IUE6 | H2AC21 | 13.99 | 52.12 | 17.69 | 1 | 3 | 3 |
| P84103 | SRSF3 | 19.32 | 51.15 | 14.02 | 1 | 2 | 2 |
| Q01844 | EWSR1 | 68.44 | 50.31 | 2.13 | 1 | 1 | 1 |
| P36957 | DLST | 48.72 | 49.33 | 1.77 | 1 | 1 | 1 |
| P0C0S5 | H2AZ1 | 13.54 | 47.24 | 14.84 | 1 | 2 | 2 |
| P16104 | H2AX | 15.14 | 47.24 | 11.89 | 1 | 2 | 2 |
| Q9P2E9 | RRBP1 | 152.36 | 47.11 | 0.43 | 1 | 1 | 1 |
| P22570 | FDXR | 53.80 | 46.23 | 1.43 | 1 | 1 | 1 |
| Q9Y5I4 | PCDHAC2 | 109.38 | 45.99 | 0.70 | 1 | 1 | 1 |
| Q9Y2X3 | NOP58 | 59.54 | 45.83 | 1.89 | 1 | 1 | 1 |
| P49137 | MAPKAPK2 | 45.54 | 45.62 | 2.75 | 1 | 1 | 1 |
| Q9BU61 | NDUFAF3 | 20.34 | 45.51 | 3.26 | 1 | 1 | 1 |
| Q6NXT2 | H3-5 | 15.20 | 45.40 | 11.85 | 2 | 2 | 2 |
| P49441 | INPP1 | 43.97 | 45.32 | 1.50 | 1 | 1 | 1 |
| P46776 | RPL27A | 16.55 | 44.72 | 7.43 | 1 | 1 | 1 |
| P05141 | SLC25A5 | 32.83 | 44.08 | 4.03 | 1 | 1 | 1 |
| P69905 | HBA1 | 15.25 | 43.78 | 6.34 | 1 | 1 | 1 |
| Q86SS6 | SYT9 | 56.15 | 43.60 | 1.43 | 1 | 1 | 1 |
| P09874 | PARP1 | 113.01 | 43.30 | 0.99 | 1 | 1 | 1 |
| P01857 | IGHG1 | 36.08 | 42.81 | 4.55 | 2 | 2 | 2 |
| P61247 | RPS3A | 29.93 | 42.56 | 3.41 | 1 | 1 | 1 |
| O00567 | NOP56 | 66.01 | 42.21 | 1.85 | 1 | 1 | 1 |
| P62424 | RPL7A | 29.98 | 41.99 | 6.39 | 2 | 2 | 2 |
| P26599 | PTBP1 | 57.19 | 41.97 | 1.51 | 1 | 1 | 1 |
| P22087 | FBL | 33.76 | 41.75 | 11.53 | 3 | 3 | 3 |
| Q8IWZ3 | ANKHD1 | 269.29 | 41.72 | 0.28 | 1 | 1 | 1 |
| P84090 | ERH | 12.25 | 41.59 | 10.58 | 1 | 1 | 1 |
| Q96LR2 | LURAP1 | 25.79 | 41.00 | 2.93 | 1 | 1 | 1 |
| P14678 | SNRPB | 24.59 | 40.63 | 6.25 | 2 | 2 | 2 |
| P46013 | MKI67 | 358.47 | 40.32 | 0.40 | 1 | 1 | 1 |
| Q9BXX3 | ANKRD30A | 158.74 | 39.88 | 0.50 | 1 | 1 | 1 |
| P26368 | U2AF2 | 53.47 | 39.59 | 1.68 | 1 | 1 | 1 |
| Q8TDQ0 | HAVCR2 | 33.37 | 39.22 | 1.99 | 1 | 1 | 1 |
| P11387 | TOP1 | 90.67 | 38.68 | 2.09 | 1 | 1 | 1 |
| P46778 | RPL21 | 18.55 | 38.47 | 16.25 | 2 | 2 | 2 |
| Q96PK6 | RBM14 | 69.45 | 38.39 | 4.04 | 2 | 2 | 2 |
| P62318 | SNRPD3 | 13.91 | 38.38 | 7.14 | 1 | 1 | 1 |
| P31327 | CPS1 | 164.83 | 37.92 | 0.67 | 1 | 1 | 1 |
| O00541 | PES1 | 67.96 | 37.79 | 1.70 | 1 | 1 | 1 |
| Q58FF6 | HSP90AB4P | 58.23 | 37.18 | 1.98 | 1 | 1 | 1 |
| P27708 | CAD | 242.83 | 36.87 | 0.36 | 1 | 1 | 1 |
| P62269 | RPS18 | 17.71 | 36.82 | 5.92 | 1 | 1 | 1 |
| Q8WU68 | U2AF1L4 | 25.73 | 36.72 | 5.45 | 1 | 1 | 1 |
| P62906 | RPL10A | 24.82 | 36.33 | 3.69 | 1 | 1 | 1 |
| Q07075 | ENPEP | 109.18 | 36.28 | 0.63 | 1 | 1 | 1 |
| P28062 | PSMB8 | 30.33 | 36.27 | 1.81 | 1 | 1 | 1 |
| Q96HU8 | DIRAS2 | 22.47 | 35.69 | 5.53 | 1 | 1 | 1 |
| Q5TB80 | CEP162 | 161.84 | 35.40 | 0.43 | 1 | 1 | 1 |
| Q86VP6 | CAND1 | 136.29 | 35.37 | 0.49 | 1 | 1 | 1 |
| Q9NUY8 | TBC1D23 | 78.27 | 34.99 | 1.14 | 1 | 1 | 1 |
| P0DOY2 | IGLC2 | 11.29 | 34.84 | 9.43 | 1 | 1 | 1 |
| P62081 | RPS7 | 22.11 | 34.56 | 6.19 | 1 | 1 | 1 |
| P33992 | MCM5 | 82.23 | 34.00 | 0.95 | 1 | 1 | 1 |
| P61254 | RPL26 | 17.25 | 33.89 | 4.14 | 1 | 1 | 1 |
| Q9HCJ5 | ZSWIM6 | 133.39 | 33.69 | 0.82 | 1 | 1 | 1 |
| Q7Z412 | PEX26 | 33.88 | 33.13 | 1.97 | 1 | 1 | 1 |
| Q92616 | GCN1 | 292.57 | 33.11 | 0.37 | 1 | 1 | 1 |
| Q8NAB2 | KBTBD3 | 69.79 | 32.93 | 1.14 | 1 | 1 | 2 |
| P62280 | RPS11 | 18.42 | 32.89 | 4.43 | 1 | 1 | 1 |
| Q5RHP9 | ERICH3 | 168.36 | 32.82 | 0.39 | 1 | 1 | 1 |
| Q9NPF5 | DMAP1 | 52.96 | 32.78 | 1.71 | 1 | 1 | 1 |
| Q9UBX7 | KLK11 | 31.04 | 32.65 | 2.13 | 1 | 1 | 1 |
| Q8WXF1 | PSPC1 | 58.71 | 32.52 | 4.40 | 1 | 1 | 1 |
| Q8NFZ5 | TNIP2 | 48.67 | 32.50 | 1.86 | 1 | 1 | 1 |
| Q9NRG0 | CHRAC1 | 14.70 | 32.18 | 6.11 | 1 | 1 | 1 |
| Q9NQR7 | CCDC177 | 79.69 | 32.14 | 1.13 | 1 | 1 | 1 |
| Q9NP81 | SARS2 | 58.25 | 32.12 | 1.54 | 1 | 1 | 1 |
| O75367 | MACROH2A1 | 39.59 | 31.98 | 2.96 | 1 | 1 | 1 |
| Q01780 | EXOSC10 | 100.77 | 31.42 | 0.79 | 1 | 1 | 1 |
| P62910 | RPL32 | 15.85 | 31.07 | 5.19 | 1 | 1 | 1 |
| A9Z1Z3 | FER1L4 | 200.85 | 31.01 | 0.33 | 1 | 1 | 1 |

Supplementary Table3. IP_IgG_Mass_Information

| **Accession** | **Gene names** | **MW [kDa]** | **Protein score** | **Sequence coverage (%)** | **# Unique Peptides** | **# Peptides** | **# PSMs** |
| --- | --- | --- | --- | --- | --- | --- | --- |
| P35527 | KRT9 | 62.03 | 1446.23 | 55.06 | 28 | 29 | 43 |
| P35908 | KRT2 | 65.39 | 1016.25 | 55.87 | 19 | 24 | 29 |
| P04264 | KRT1 | 66.00 | 757.11 | 34.32 | 20 | 25 | 34 |
| P13645 | KRT10 | 58.79 | 755.74 | 38.87 | 18 | 21 | 28 |
| Q86YZ3 | HRNR | 282.23 | 537.47 | 16.56 | 11 | 11 | 16 |
| P05787 | KRT8 | 53.67 | 490.25 | 41.20 | 16 | 21 | 26 |
| P05783 | KRT18 | 48.03 | 371.33 | 36.74 | 14 | 16 | 17 |
| P02533 | KRT14 | 51.53 | 290.75 | 28.18 | 9 | 14 | 16 |
| P08727 | KRT19 | 44.08 | 256.74 | 35.50 | 9 | 14 | 14 |
| P60709 | ACTB | 41.71 | 203.92 | 33.87 | 11 | 11 | 14 |
| O60814 | H2BC12 | 13.88 | 192.24 | 39.68 | 5 | 5 | 10 |
| P02768 | ALB | 69.32 | 188.95 | 9.03 | 6 | 6 | 7 |
| P06748 | NPM1 | 32.55 | 171.51 | 11.56 | 2 | 2 | 3 |
| P11142 | HSPA8 | 70.85 | 165.01 | 8.36 | 2 | 4 | 4 |
| P13647 | KRT5 | 62.34 | 155.39 | 22.88 | 6 | 16 | 17 |
| Q07020 | RPL18 | 21.62 | 136.07 | 19.68 | 3 | 3 | 3 |
| P13804 | ETFA | 35.06 | 127.66 | 5.71 | 1 | 1 | 2 |
| P02538 | KRT6A | 60.01 | 120.27 | 17.91 | 2 | 12 | 13 |
| P62805 | H4C1 | 11.36 | 115.75 | 29.13 | 3 | 3 | 3 |
| P11021 | HSPA5 | 72.29 | 107.74 | 6.42 | 1 | 3 | 3 |
| Q96KK5 | H2AC12 | 13.90 | 104.44 | 32.81 | 4 | 4 | 5 |
| P68104 | EEF1A1 | 50.11 | 102.13 | 13.42 | 5 | 5 | 5 |
| P36578 | RPL4 | 47.67 | 100.68 | 7.96 | 3 | 3 | 3 |
| P32119 | PRDX2 | 21.88 | 99.44 | 14.14 | 3 | 3 | 3 |
| P62899 | RPL31 | 14.45 | 94.20 | 18.40 | 2 | 2 | 2 |
| A0A075B6S2 | IGKV2D-29 | 13.13 | 92.15 | 16.67 | 2 | 2 | 2 |
| P25705 | ATP5F1A | 59.71 | 86.01 | 2.35 | 1 | 1 | 2 |
| P62263 | RPS14 | 16.26 | 82.12 | 7.28 | 1 | 1 | 1 |
| P04406 | GAPDH | 36.03 | 76.57 | 4.18 | 1 | 1 | 1 |
| O00148 | DDX39A | 49.10 | 76.19 | 2.34 | 1 | 1 | 1 |
| Q02878 | RPL6 | 32.71 | 72.52 | 8.33 | 2 | 2 | 2 |
| P35579 | MYH9 | 226.39 | 70.82 | 1.94 | 3 | 3 | 3 |
| P62266 | RPS23 | 15.80 | 69.41 | 7.69 | 1 | 1 | 1 |
| P07437 | TUBB | 49.64 | 69.18 | 9.91 | 2 | 2 | 2 |
| P47914 | RPL29 | 17.74 | 66.26 | 9.43 | 1 | 1 | 1 |
| Q6NVV1 | RPL13AP3 | 12.13 | 65.86 | 10.78 | 1 | 1 | 1 |
| P16403 | H1-2 | 21.35 | 63.10 | 16.90 | 1 | 3 | 3 |
| P61353 | RPL27 | 15.79 | 61.88 | 12.50 | 2 | 2 | 2 |
| P46776 | RPL27A | 16.55 | 60.55 | 7.43 | 1 | 1 | 1 |
| P20930 | FLG | 434.92 | 60.01 | 0.20 | 1 | 1 | 1 |
| P18124 | RPL7 | 29.21 | 59.57 | 4.44 | 1 | 1 | 1 |
| P06312 | IGKV4-1 | 13.37 | 59.53 | 13.22 | 2 | 2 | 2 |
| P38646 | HSPA9 | 73.63 | 58.65 | 3.09 | 2 | 2 | 2 |
| P62917 | RPL8 | 28.01 | 56.92 | 4.28 | 1 | 1 | 1 |
| P26373 | RPL13 | 24.25 | 54.70 | 22.27 | 5 | 5 | 5 |
| A0A075B6I1 | IGLV4-60 | 12.98 | 54.47 | 8.33 | 1 | 1 | 1 |
| O76031 | CLPX | 69.18 | 53.15 | 2.05 | 1 | 1 | 1 |
| P36957 | DLST | 48.72 | 51.20 | 1.77 | 1 | 1 | 1 |
| Q9Y5I4 | PCDHAC2 | 109.38 | 49.46 | 0.70 | 1 | 1 | 1 |
| B2RXH8 | HNRNPCL2 | 32.05 | 49.11 | 3.07 | 1 | 1 | 1 |
| P10412 | H1-4 | 21.85 | 48.63 | 20.55 | 1 | 4 | 4 |
| P49441 | INPP1 | 43.97 | 48.37 | 1.50 | 1 | 1 | 1 |
| P42766 | RPL35 | 14.54 | 48.13 | 8.13 | 1 | 1 | 1 |
| P01624 | IGKV3-15 | 12.49 | 47.87 | 7.83 | 1 | 1 | 1 |
| A4FU01 | MTMR11 | 79.50 | 46.99 | 0.99 | 1 | 1 | 1 |
| Q9P2E9 | RRBP1 | 152.36 | 46.86 | 0.43 | 1 | 1 | 1 |
| Q8IVT2 | MISP | 75.31 | 46.79 | 1.62 | 1 | 1 | 1 |
| P81605 | DCD | 11.28 | 46.05 | 10.00 | 1 | 1 | 1 |
| P68431 | H3C1 | 15.39 | 45.31 | 16.91 | 3 | 3 | 3 |
| P83731 | RPL24 | 17.77 | 44.04 | 8.28 | 1 | 1 | 1 |
| Q32P51 | HNRNPA1L2 | 34.20 | 43.20 | 2.81 | 1 | 1 | 1 |
| P62424 | RPL7A | 29.98 | 42.28 | 2.26 | 1 | 1 | 1 |
| P62913 | RPL11 | 20.24 | 42.17 | 5.06 | 1 | 1 | 1 |
| P27708 | CAD | 242.83 | 40.28 | 0.36 | 1 | 1 | 1 |
| P20674 | COX5A | 16.75 | 39.99 | 4.00 | 1 | 1 | 2 |
| P15880 | RPS2 | 31.30 | 39.94 | 2.39 | 1 | 1 | 1 |
| Q8TDQ0 | HAVCR2 | 33.37 | 39.52 | 1.99 | 1 | 1 | 1 |
| P20700 | LMNB1 | 66.37 | 39.46 | 1.71 | 1 | 1 | 1 |
| P06733 | ENO1 | 47.14 | 39.42 | 1.38 | 1 | 1 | 1 |
| Q9Y6R1 | SLC4A4 | 121.38 | 39.12 | 0.65 | 1 | 1 | 1 |
| Q96HU8 | DIRAS2 | 22.47 | 38.45 | 5.53 | 1 | 1 | 1 |
| Q07075 | ENPEP | 109.18 | 37.98 | 0.63 | 1 | 1 | 1 |
| P62277 | RPS13 | 17.21 | 37.31 | 4.64 | 1 | 1 | 1 |
| Q00839 | HNRNPU | 90.53 | 37.27 | 2.18 | 1 | 1 | 1 |
| Q8NAB2 | KBTBD3 | 69.79 | 36.62 | 1.14 | 1 | 1 | 1 |
| Q6ZMV7 | LEKR1 | 45.13 | 36.41 | 1.80 | 1 | 1 | 1 |
| P31327 | CPS1 | 164.83 | 36.40 | 0.67 | 1 | 1 | 1 |
| P11498 | PC | 129.55 | 36.21 | 0.85 | 1 | 1 | 1 |
| Q8IWZ3 | ANKHD1 | 269.29 | 36.11 | 0.28 | 1 | 1 | 1 |
| Q7Z412 | PEX26 | 33.88 | 36.06 | 1.97 | 1 | 1 | 1 |
| P22570 | FDXR | 53.80 | 35.87 | 1.43 | 1 | 1 | 1 |
| P62249 | RPS16 | 16.44 | 35.35 | 6.85 | 1 | 1 | 1 |
| Q8NEE6 | FBXL13 | 83.87 | 34.59 | 0.95 | 1 | 1 | 1 |
| P49643 | PRIM2 | 58.77 | 33.87 | 1.18 | 1 | 1 | 1 |
| Q8TCS8 | PNPT1 | 85.90 | 33.63 | 0.77 | 1 | 1 | 1 |
| P0DOY2 | IGLC2 | 11.29 | 33.58 | 9.43 | 1 | 1 | 1 |
| P19338 | NCL | 76.57 | 33.48 | 2.54 | 2 | 2 | 2 |
| O95292 | VAPB | 27.21 | 33.40 | 2.88 | 1 | 1 | 1 |
| Q12934 | BFSP1 | 74.50 | 32.98 | 1.05 | 1 | 1 | 1 |
| Q13424 | SNTA1 | 53.86 | 32.54 | 1.39 | 1 | 1 | 1 |
| Q9NRG0 | CHRAC1 | 14.70 | 32.43 | 6.11 | 1 | 1 | 1 |
| Q9NUY8 | TBC1D23 | 78.27 | 32.35 | 1.14 | 1 | 1 | 1 |
| P46781 | RPS9 | 22.58 | 32.31 | 4.12 | 1 | 1 | 1 |
| P01699 | IGLV1-44 | 12.19 | 32.31 | 5.98 | 1 | 1 | 1 |
| Q9NQR7 | CCDC177 | 79.69 | 32.21 | 1.13 | 1 | 1 | 1 |
| Q8WYA0 | IFT81 | 79.70 | 32.10 | 0.89 | 1 | 1 | 1 |
| P00966 | ASS1 | 46.50 | 31.99 | 1.70 | 1 | 1 | 1 |
| Q2M3G4 | SHROOM1 | 90.73 | 31.91 | 1.17 | 1 | 1 | 1 |
| O15105 | SMAD7 | 46.40 | 31.67 | 2.35 | 1 | 1 | 1 |
| Q96M86 | DNHD1 | 533.30 | 31.62 | 0.13 | 1 | 1 | 1 |
| Q8TDN4 | CABLES1 | 67.56 | 31.62 | 1.11 | 1 | 1 | 1 |
| P46779 | RPL28 | 15.74 | 31.56 | 8.03 | 1 | 1 | 1 |
| Q8TB33 | LINC01560 | 10.35 | 31.24 | 8.51 | 1 | 1 | 1 |
| P61254 | RPL26 | 17.25 | 31.11 | 4.14 | 1 | 1 | 1 |
| Q8NFZ5 | TNIP2 | 48.67 | 30.88 | 1.86 | 1 | 1 | 1 |
| P62241 | RPS8 | 24.19 | 30.74 | 6.25 | 1 | 1 | 1 |
| P11047 | LAMC1 | 177.49 | 30.73 | 0.44 | 1 | 1 | 1 |
| P01286 | GHRH | 12.44 | 30.61 | 7.41 | 1 | 1 | 1 |
